# Differential maturation and chaperone dependence of the paralogous protein kinases DYRK1A and DYRK1B

**DOI:** 10.1038/s41598-022-06423-0

**Published:** 2022-02-14

**Authors:** Marco Papenfuss, Svenja Lützow, Gerrit Wilms, Aaron Babendreyer, Maren Flaßhoff, Conrad Kunick, Walter Becker

**Affiliations:** 1 Institute of Pharmacology and Toxicology, RWTH Aachen University, Wendlingweg 2, 52074 Aachen, Germany; 2 Institute of Molecular Pharmacology, RWTH Aachen University, 52074 Aachen, Germany; 3 Institut für Medizinische und Pharmazeutische Chemie, Technische Universität Braunschweig, 38106 Braunschweig, Germany

**Keywords:** Biochemistry, Biological techniques, Structural biology

## Abstract

The HSP90/CDC37 chaperone system not only assists the maturation of many protein kinases but also maintains their structural integrity after folding. The interaction of mature kinases with the HSP90/CDC37 complex is governed by the conformational stability of the catalytic domain, while the initial folding of the protein kinase domain is mechanistically less well characterized. DYRK1A (Dual-specificity tyrosine (Y)-phosphorylation Regulated protein Kinase 1A) and DYRK1B are closely related protein kinases with discordant HSP90 client status. DYRK kinases stoichiometrically autophosphorylate on a tyrosine residue immediately after folding, which served us as a traceable marker of successful maturation. In the present study, we used bacterial expression systems to compare the capacity of autonomous maturation of DYRK1A and DYRK1B in the absence of eukaryotic cofactors or chaperones. Under these conditions, autophosphorylation of human DYRK1B was severely compromised when compared with DYRK1A or DYRK1B orthologs from zebrafish and Xenopus. Maturation of human DYRK1B could be restored by bacterial expression at lower temperatures, suggesting that folding was not absolutely dependent on eukaryotic chaperones. The differential folding properties of DYRK1A and DYRK1B were largely due to divergent sequences of the C-terminal lobes of the catalytic domain. Furthermore, the mature kinase domain of DYRK1B featured lower thermal stability than that of DYRK1A when exposed to heat challenge in vitro or in living cells. In summary, our study enhances the mechanistic understanding of the differential thermodynamic properties of two closely related protein kinases during initial folding and as mature kinases.

## Introduction

Dual-specificity tyrosine phosphorylation-regulated kinases (DYRKs) constitute a family of protein kinases within the CMGC group of kinases, which includes cyclin-dependent kinases (CDKs), mitogen-activated kinases (MAPKs), glycogen synthase kinases (GSK) and CDK-like kinases (CLKs). DYRKs are divided into two classes based on their phylogenetic relationship and structural organization^1^. Class 1 DYRKs are distinguished from class 2 DYRKs by the conserved structure of their N-terminal region, which harbors a bipartite nuclear localization signal and a binding site for the adaptor protein DCAF7^2^. Two paralogous genes encode the mammalian class 1 DYRKs, DYRK1A and DYRK1B (also known as Mirk^3^). DYRK1A and DYRK1B have similar functions in cell cycle control and share several substrates^4,5^. However, DYRK1A and DYRK1B have different patterns of expression, and the divergent sequences of their C-terminal domains result in different subcellular localization and protein–protein interactions^6,7^.

DYRKs phosphorylate their substrates on serine or threonine residues but autophosphorylate a tyrosine residue in the activation loop of the catalytic domain^8–11^. This intramolecular autophosphorylation is a one-time event and is catalyzed by a folding intermediate that is structurally distinct from the mature conformation. Tyrosine autophosphorylation (Tyr321 in DYRK1A, Tyr273 in DYRK1B) is essential for full catalytic activity of all DYRKs and the related homeodomain-interacting protein kinases (HIPKs)^3,8,10,12–14^. Still during maturation, DYRK1A subsequently autophosphorylates on Ser97 in the N-terminal region, which prevents the degradation of the protein^15^.

The capacity of auto-activation is an intrinsic property of the catalytic domain of DYRK1A and can take place in the absence of the non-catalytic domains or any other assisting protein such as chaperones^11^. In contrast, human DYRK1B does not autophosphorylate on tyrosine when expressed in a cell-free bacterial in vitro-translation system^11^. Furthermore, pharmacological inhibition of the HSP90 chaperone function prevented the activating autophosphorylation of DYRK1B but not DYRK1A when expressed in mammalian cell lines^16^. In addition, HSP90 inhibitors induce rapid aggregation and long-term depletion of DYRK1B without affecting DYRK1A^17^. Protein interaction assays revealed that DYRK1B but not DYRK1A is associated with HSP90 and the co-chaperone CDC37^16,17^.

What is the structural basis for the differential chaperone dependence of these closely related kinases? DYRK1A and DYRK1B share 85% sequence identity in the catalytic domain, and molecular models of DYRK1B based on DYRK1A structures did not reveal apparent differences in the hypothetical structure of the kinase domain^16,18^. All residues involved in ATP binding and catalysis as well as the sequence of the activation loop with the autophosphorylated tyrosine are identical in DYRK1A and DYRK1B. However, DYRK1B harbors three unique residues N-terminal of the catalytic domain that are not found in other class 1 DYRKs (supplementary Fig. S1). These amino acids are located in a conserved sequence designated DYRK homology (DH) box^19^. The DH box is essential for tyrosine autophosphorylation of the *Drosophila* dDYRK2 kinase^20^. We have recently shown that two residues in the DH box are critical for the maturation of DYRK1B and DYRK1A^16,21^. Crystallization of DYRK1A and DYRK2 revealed that the DH box stabilizes the kinase domain through a large network of interactions^22^. It is not yet known whether the differences in the DH box sequence are responsible for the differential folding properties and chaperone dependence of DYRK1A and DYRK1B.

HSP90-interacting kinases do not share conserved sequence determinants. Rather, the extent of client binding by HSP90 is governed by the thermodynamic stability of the catalytic domain^23,24^. The conformational instability allows the HSP90-CDC37 complex to interact with a partially unfolded state of the mature kinase domain^25^. Similar principles are thought to underlie the function of HSP90/CDC37 in the initial folding of protein kinases^25^. However, it is difficult to experimentally distinguish the chaperone dependence of post-translational folding from the stabilization of the mature kinase domain.

The present study aims to delineate the structural features that result in the discordant chaperone dependence of the initial folding of DYRK1A and DYRK1B as well as the thermal stability of the mature catalytic domain. We employed bacterial expression as a model system for chaperon-independent maturation since prokaryotic HSP90 does not assist eukaryotic protein kinases^26^. The phosphotyrosine content of DYRKs can be exploited as a faithful marker of productive posttranslational folding since it is not altered by later misfolding due to thermodynamic instability of the mature kinase domain. With the help of chimeric DYRK1A/DYRK1B constructs, we identified the C-terminal lobe (C-lobe) of the catalytic domain as the major factor that accounts for the impaired maturation of DYRK1B in the absence of chaperones. Furthermore, we provide experimental evidence that the mature catalytic domain of DYRK1B features lower thermal stability than DYRK1A.

## Results

**Figure 1:**
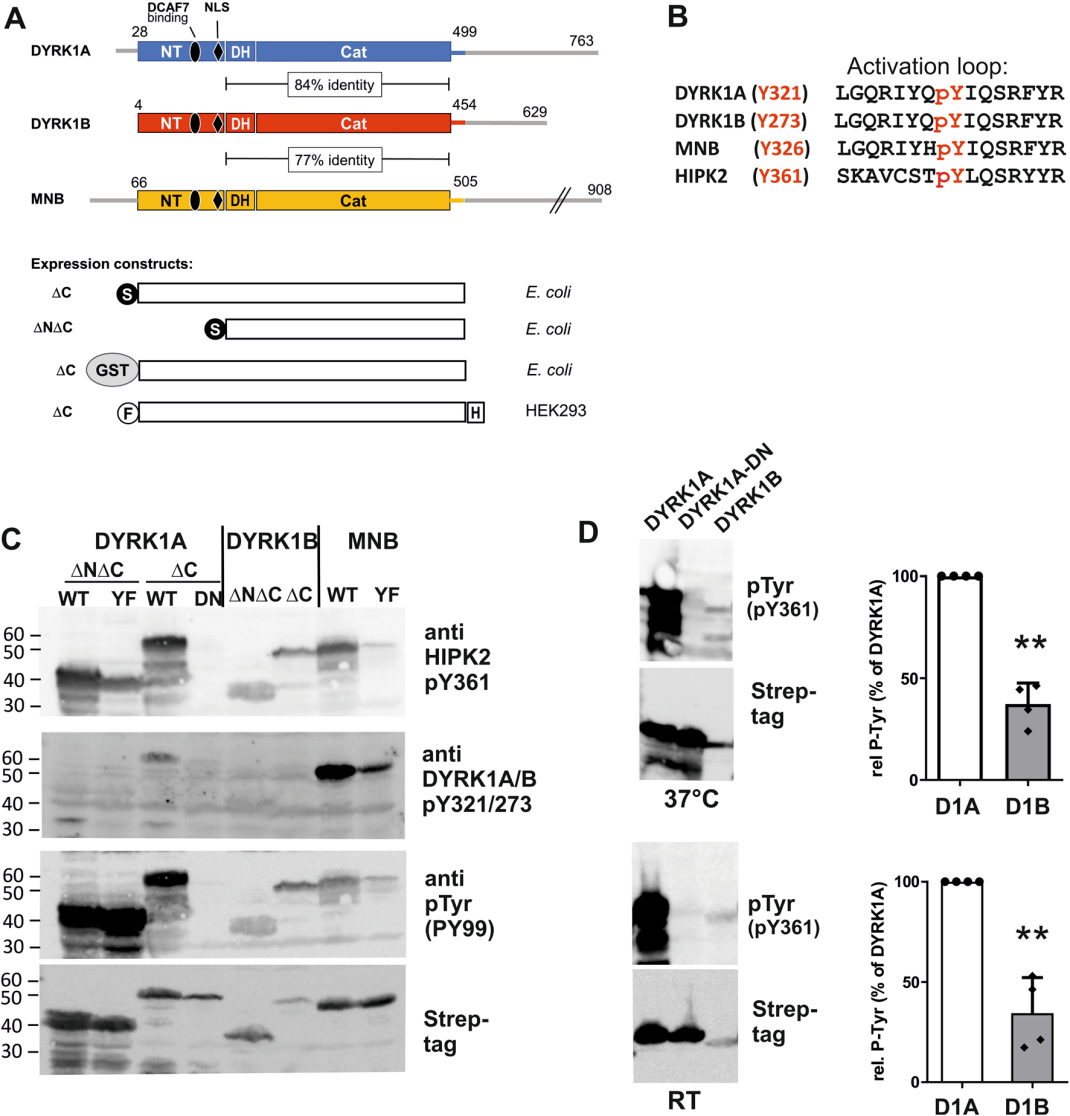
Detection of tyrosine autophosphorylation in DYRK1A, DYRK1B and MNB. (**A**) Domain structure of selected class 1 DYRKs and design of expression constructs. Conserved sequence regions include the catalytic domain (cat), the DH box and the N-terminal region (NT), which harbors a binding site for the adaptor protein DCAF7 and a bipartite nuclear localization signal (NLS). Expression constructs employed in the present study (designated ΔC) were fused to a Strep-tag (S), glutathione S-transferase (GST), or the FLAG (F) and HiBiT (H) tags. The shorter constructs (ΔNΔC) have been described previously^11^. The exact boundaries of the different subdomains are listed in the supplementary information. (**B**) Sequence surrounding the autophosphorylated tyrosine in the activation loop. The alignment illustrates the structural basis for the cross-reaction of the HIPK2(pY361) antibody with class 1 DYRKs. (**C**) Detection of tyrosine autophosphorylation in *E. coli*. The indicated deletion constructs of rat DYRK1A, human DYRK1B and *Drosophila* MNB were expressed in *E. coli* for 3 h at 37 °C. Total cell lysates were subjected to immunoblot analysis with different commercial antibodies that were directed against the tyrosine in the activation loop of HIPK2 (pY361) or DYRK1A/B (pY321/273), or with an antibody that detects phosphotyrosine (pTyr) independent of the sequence context (PY99). Total recombinant protein was visualized by detection of the N-terminal Strep-tag. Point mutants used as controls include a catalytically inactive point mutant of DYRK1A (DN, D287N) and mutants of the autophosphorylation site in the activation loop (YF, Y321F in DYRK1A and Y326F in MNB). (**D**) Comparison of DYRK1A and DYRK1B. DYRK1-ΔC constructs were expressed in *E. coli* for 3 h at 37 °C or for 20 h at room temperature (20–25 °C). Tyrosine autophosphorylation was detected by immunoblot analysis of total bacterial lysates with the pY361(HIPK2) antibody. Relative phosphorylation was densitometrically quantitated (ratio of pTyr to Strep-tag signal). The graphs illustrate the results of n = 4 experiments (means and SD; One sample t test).

The catalytic domain of DYRK1A, together with the adjacent DH box, can fold autonomously and attain its active conformation, but N-terminal sequences are relevant for the maturation and stability of several DYRKs^15,20,27^. Therefore, we generated new expression constructs of mammalian DYRK1A, DYRK1B, and their *Drosophila* ortholog *minibrain* (MNB)^28^ that included the conserved N-terminal regions of these kinases (Fig. 1A). The variable C-terminal regions were excluded to avoid potential effects unrelated to maturation and conformational stability of the catalytic domain. These constructs were expressed in *E. coli* to explore their capacity of tyrosine autophosphorylation in the absence of eukaryotic chaperones or other co-factors. For comparison, we also included our previous deletion constructs that lack the N-terminal region^11^ (ΔNΔC in Fig. 1A). Different antibodies were used to assess the phosphotyrosine content of the DYRK1 constructs by immunoblot analysis. PY99 is a monoclonal antibody that detects phosphorylated tyrosines independent of the sequence context. Two polyclonal antibodies are commercially marketed for detection of the phosphotyrosine in DYRK1A (pY321) and DYRK1B (pY273) or HIPK2 (pY361), which share closely related sequences in the activation loop (Fig. 1B).

Figure 1C shows that the new expression constructs (ΔC) are expressed as active kinases in *E. coli*, as evidenced by the detection of tyrosine autophosphorylation. The presence or absence of the N-terminal region had no major effect on the level of phosphotyrosine in DYRK1A or DYRK1B.

We and others have previously utilized the pY361(HIPK2) antibody to detect the autophosphorylation of DYRK1A and DYRK1B^16,21,29^. However, this antibody recognized DYRK1A-Y321F, indicating that it is not absolutely specific for the activating autophosphorylation site. The purported “phospho-DYRK1A/DYRK1B (Tyr321, Tyr273)” antibody was better suited to detect tyrosine-phosphorylated MNB rather than DYRK1A/B (Fig. 1C). Therefore, the phospho-HIPK2(Tyr361) antibody was used in all further experiments to detect the autophosphorylation of DYRK1 constructs. Figure 1D shows that DYRK1B is not only expressed at lower levels but also contains less phosphotyrosine than DYRK1A, suggesting that the initial folding is impaired. This difference persisted when the DYRK1 constructs were expressed at room temperature. The low expression of DYRK1B is unlikely due to the usage of rare codons, since codon usage analysis yielded a higher *E. coli* codon adaptation index (CAI) for DYRK1B than for DYRK1A (see supplementary methods).

### Maturation and functional integrity of DYRK1B is temperature-sensitive

**Figure 2:**
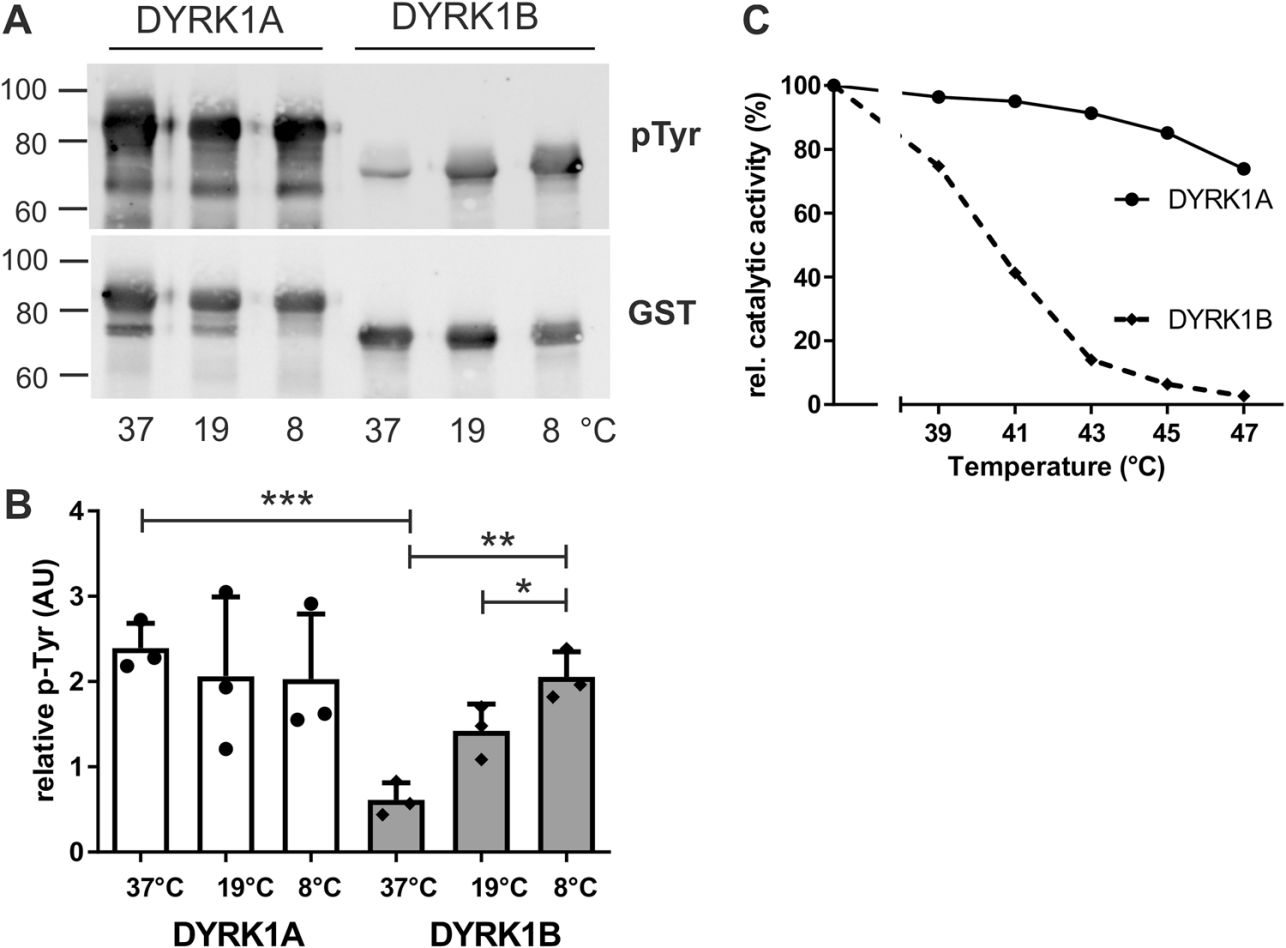
Temperature dependence of GST-DYRK1B maturation and stability. (**A**,**B**) Temperature effects on maturation of the catalytic domain. GST-DYRK1AΔC and GST-DYRK1BΔC were expressed in *E. coli* at 37 °C (4 h), 19 °C (23 h) or 8 °C (48 h). GST fusion proteins were partially purified by affinity adsorption to glutathione Sepharose before Western blot analysis (**A**). Panel (**B**) shows the quantitative evaluation of n = 3 experiments (means and SD). (**C**) Thermal stability of the mature kinase domain. Samples of GST-DYRK1AΔC and GST-DYRK1BΔC with similar catalytic activity were heat challenged for 3.5 min at variable temperatures before the proteins were subjected to a kinase assay with a peptide substrate. Catalytic activities were normalized to the activity of a control sample that was not exposed to heat shock. This result was reproduced with independent preparations of GST-DYRK1A and GST-DYRK1B (supplementary Fig. S2). *AU* arbitrary units.

As we have previously produced active DYRK1B as a fusion protein with glutathione S-transferase (GST)^16^, we used this system to compare tyrosine autophosphorylation of C-terminally deleted GST-DYRK1A and GST-DYRK1B. We mused that the N-terminal GST can promote solubility of fusion proteins in *E. coli* and reduce aggregation of immature, unfolded protein^30^. Furthermore, the pGEX vector does not use the very strong T7 polymerase promoter that drives the expression of the Strep-Tag constructs in the pET vector. As shown in Fig. 2, GST-DYRK1B contained much lower levels of phosphotyrosine than GST-DYRK1A when expressed at 37 °C. However, autophosphorylation was enhanced when GST-DYRK1B was expressed at 19 °C or 8 °C. Cultivation at reduced temperatures slows down the rate of protein synthesis and folding kinetics and may thereby decrease the hydrophobic interactions that result in misfolding. These results show that maturation of DYRK1B in the absence of eukaryotic chaperones is possible but is thermodynamically more vulnerable than DYRK1A.

Next we tested whether DYRK1A and DYRK1B also differed in their thermal stability after maturation (Fig. 2C). Purified preparations of GST-DYRK1A and GST-DYRK1B were matched for similar catalytic activity and subjected to a 3.5 min heat shock before the functional integrity of the catalytic domain was assessed by means of a kinase assay. A short heat treatment at 43 °C largely inactivated DYRK1B, while DYRK1A retained most of its activity at this temperature.

### DYRK1A and DYRK1B in different vertebrates

**Figure 3:**
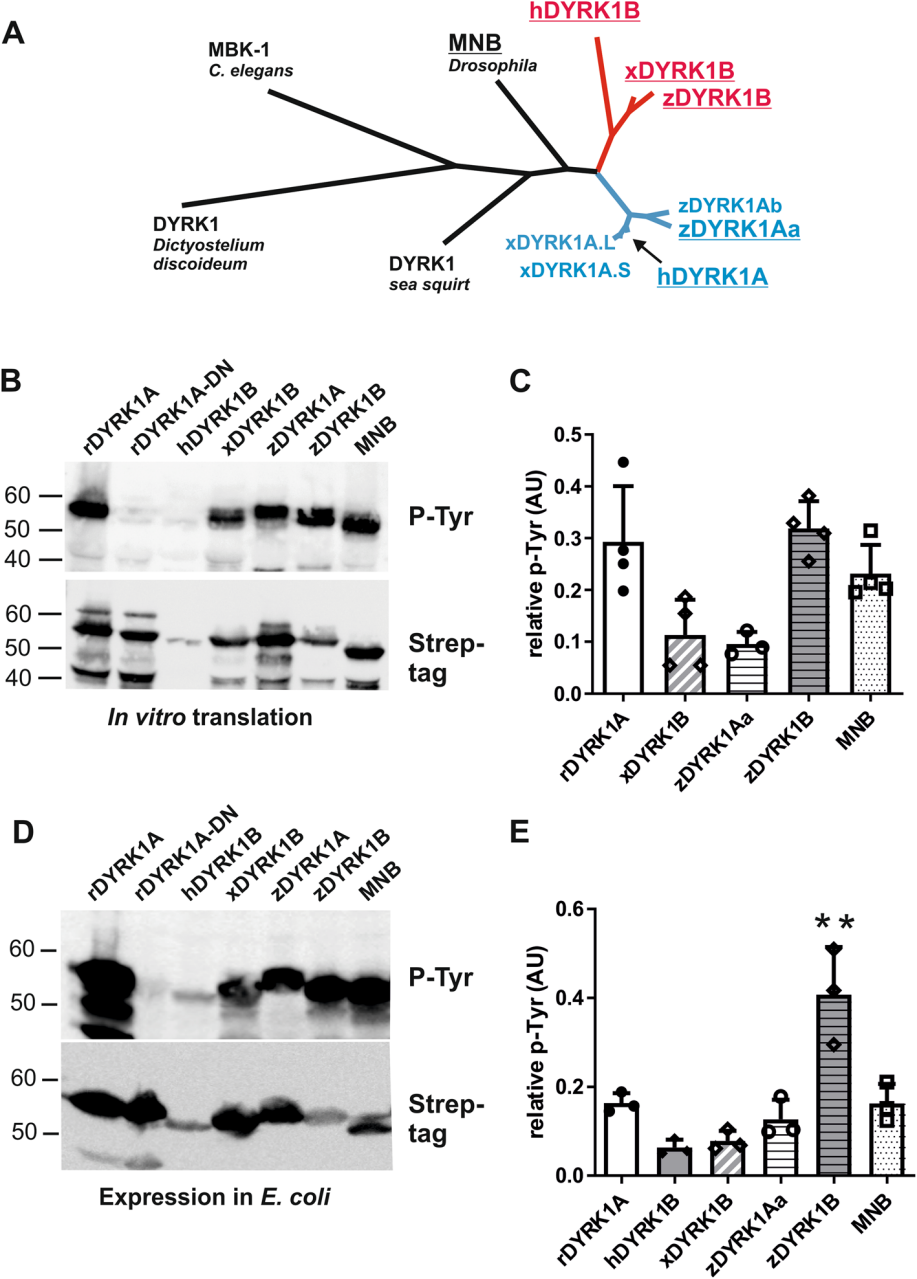
Tyrosine autophosphorylation of different vertebrate class 1 DYRKs. (**A**) Dendrogram of selected class 1 DYRKs. Invertebrates harbor one class I DYRK while most vertebrates contain the paralogous DYRK1A (blue) and DYRK1B (red). Genome duplications resulted in the presence of two DYRK1A genes in Zebrafish (*Danio rerio*) and *Xenopus laevis*. Sequence similarity is indicated by branch lengths. The DYRK1 proteins characterized below are underlined. The rat DYRK1A construct differs by a single amino acid from the human sequence. (**B**,**C**) Cell free expression. The indicated class 1 DYRKs from rat (r), human (h), *Xenopus* (x), zebrafish (z) or *Drosophila* (MNB) were expressed by coupled in vitro transcription and translation for 2 h at 37 °C. The catalytically inactive mutant of DYRK1A (DYRK1A-D287N) was included as a technical control to monitor the background signal of the unphosphorylated kinase. Tyrosine autophosphorylation was detected by immunoblot analysis of total cellular lysates and relative phosphorylation was quantitated by densitometric evaluation (ratio of p-Tyr to Strep-tag signal). hDYRK1B values are not shown because signal intensities were too close to background levels. The scatter plot shows the means and SD of n = 4 replicate experiments (except for zDYRK1Aa, n = 3). (**D**,**E**) Expression in *E. coli*. Expression of the indicated class 1 DYRKs proceeded for 3 h at 37 °C. Tyrosine autophosphorylation of the recombinant kinases was assessed by immunoblot analysis of total bacterial lysates (**D**). Panel E illustrates the results of n = 3 replicate experiments (means and SD).

DYRK1A and DYRK1B originated by gene duplication and exist as paralogous genes in jawed vertebrates except for cartilaginous fish (Fig. 3A). MNB and DYRK1A, but not human DYRK1B, are capable of autonomous maturation by tyrosine autophosphorylation when expressed by cell-free translation at 37 °C^9,11^. We asked whether the maturation of DYRK1B orthologs from other vertebrate classes was also compromised in the absence of eukaryotic chaperones. To this end, we chose DYRK1B from zebrafish (*Danio rerio*)^31^ and African clawed frog (*Xenopus laevis*)^32^ as representatives for bony fish and amphibians, and included zebrafish DYRK1Aa^33^ for further comparison. Strep-tag fusion constructs were designed as illustrated in Fig. 1A (containing only the catalytic domain and the conserved N-terminal region).

The expression plasmids were used as templates for coupled in vitro transcription and translation in a cell-free expression system reconstituted from purified components necessary for *E. coli* translation. All tested kinases except human DYRK1B contained well detectable levels of phosphotyrosine (Fig. 3B,C). This result supports the hypothesis that the capacity of chaperone-independent tyrosine autophosphorylation is an ancestral characteristic of class 1 DYRKs. As shown in Fig. 1C, the P-Tyr signal does not strictly prove autophosphorylation of the tyrosine in the activation loop but can be due to other tyrosines that may vary between the different DYRK1 orthologs. Nevertheless, phosphotyrosine content depends on the catalytic activity and thus reflects the correct folding of the kinase domain.

Human DYRK1B was expressed at much lower levels than the other constructs, which hampered the quantitative evaluation of relative autophosphorylation. Similar results were obtained when the constructs were expressed in *E. coli* cells (Fig. 3D,E). The highest levels of phosphotyrosine were detected in bacterially expressed DYRK1B from zebrafish. Thus, the unproductive expression and maturation of human DYRK1B in *E. coli* is not a universal feature of vertebrate DYRK1B orthologs. Notably, human DYRK1B is most divergent from the last common ancestor of vertebrate class 1 DYRKs (Fig. 3A, supplementary Fig. S1).

### Dissecting the structural elements required for maturation of class 1 DYRKs

**Figure 4:**
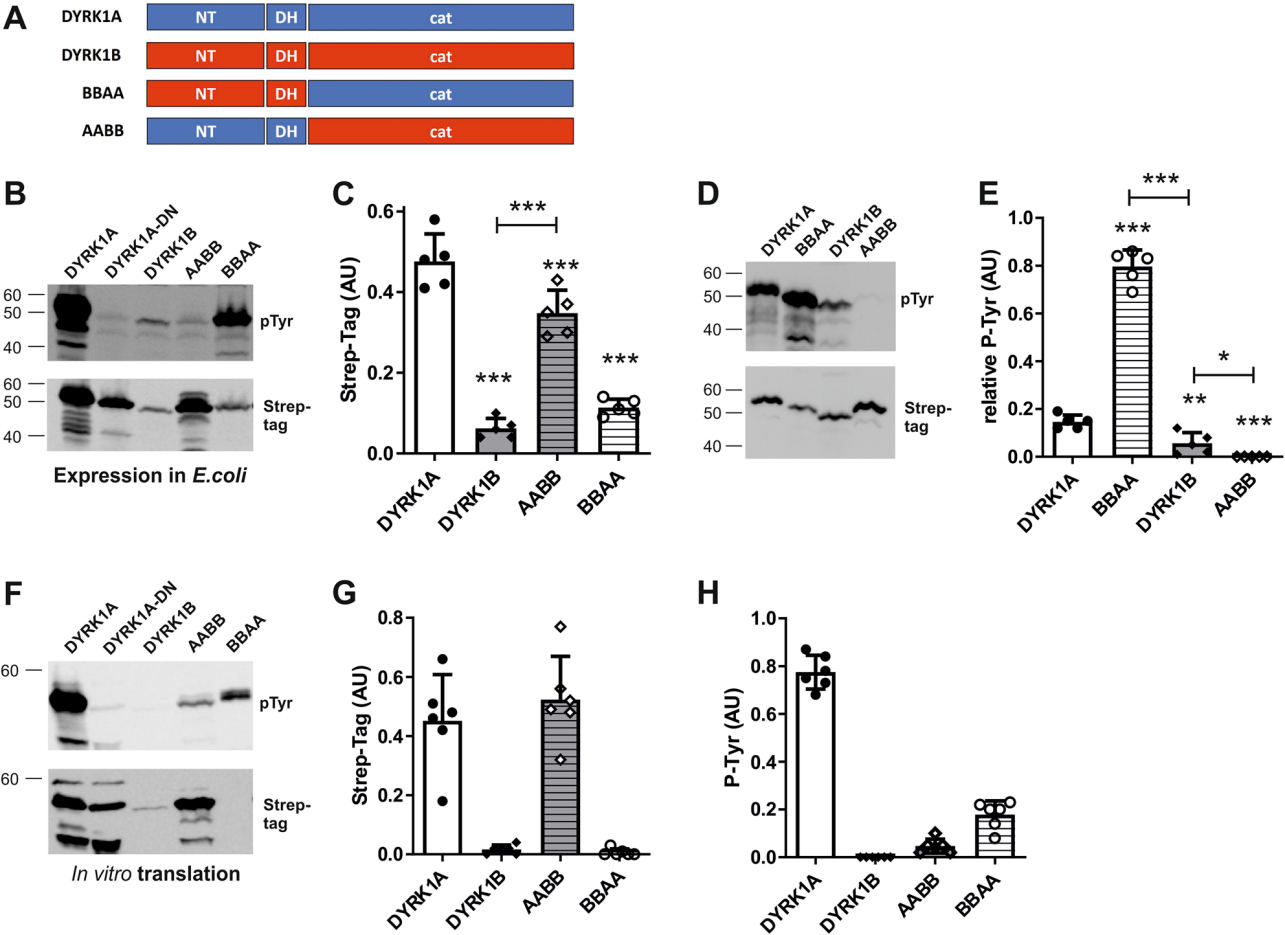
Expression and autophosphorylation of chimeric DYRK1A/DYRK1B constructs. (**A**) Scheme of the chimeric constructs. The designations of the chimeras indicate whether the N-terminal region (NT, first letter), DH box (DH, second letter) or N-terminal and C-terminal part of the catalytic domain (cat, third and fourth letter) originate from DYRK1A (blue) or DYRK1B (red). Information on the amino acid positions of the different sequence parts can be found in the supplementary information. (**B**–**E**) Bacterial expression. Tyrosine autophosphorylation of bacterially expressed kinases (37 °C) was detected by Western blot analysis. DYRK1A-D287N served as background control. Panel C shows the expression levels of the different constructs as determined by densitometric evaluation of the Strep-tag signals (means and SD, n = 5, including the experiments presented in Fig. 5C). To allow the quantitative evaluation of tyrosine autophosphorylation of constructs with strongly different expression levels, sample volumes were adjusted to better match the amounts of the recombinant proteins on the Western blot (**D**). Panel (**E**) illustrates the results of 5 independent experiments (means and SD). Unless otherwise indicated, statistical significance is given for the comparison with DYRK1A. (**F**–**H**) Cell-free expression. Constructs were expressed for 2 h at 37 °C by in vitro translation. The graphs illustrate expression levels (**G**) and phosphotyrosine signals (**H**) (means and SD, n = 6; including the experiments presented in Fig. 5F). Relative autophosphorylation was not calculated and results were not statistically evaluated because signal intensities of DYRK1B and BBAA were too close to background levels.

Next we asked whether the N-terminal region, together with the DH box, or the catalytic domain accounts for the different properties of the Strep-tag DYRK1A and DYRK1B constructs. Therefore, we designed chimeric constructs of DYRK1A and DYRK1B in which their N-terminal and catalytic regions were swapped for the respective counterparts from the other kinase (Fig. 4A). The chimeric constructs were expressed in *E. coli* cells and by in vitro translation to analyze their expression levels and capacity to attain the active, autophosphorylated state in the absence of eukaryotic chaperones.

In both expression systems, DYRK1A and the AABB chimera were produced to higher levels than DYRK1B and BBAA (Fig. 4B,C,F,G), indicating that the N-terminal region accounts for the different expression levels of DYRK1A and DYRK1B*.* In contrast, the lower level of phosphotyrosine in DYRK1B can be attributed to the catalytic domain (Fig. 4D,E, compare DYRK1A with AABB and DYRK1B with BBAA). Unexpectedly, tyrosine autophosphorylation of the DYRK1A catalytic domain was markedly increased by fusion to the N-terminal region of DYRK1B (DYRK1A *vs*. BBAA), while the autophosphorylation of DYRK1B was further reduced when its N-terminal region was substituted by that of DYRK1A (DYRK1B vs. AABB; Fig. 4E). Still, DYRK1A contained more phosphotyrosine than DYRK1B (Fig. 4E), indicating that the positive impact of the catalytic domain of DYRK1A on tyrosine autophosphorylation exceeded that of the N-terminal region of DYRK1B. Similar results were observed in the cell-free expression system, although the weak expression of DYRK1B and BBAA precluded statistical evaluation (Fig. 4G,H). These results support the conclusion that the impaired maturation of DYRK1B is a property of the catalytic domain itself.

### Impact of the C-terminal lobe of the catalytic domain on tyrosine autophosphorylation

**Figure 5:**
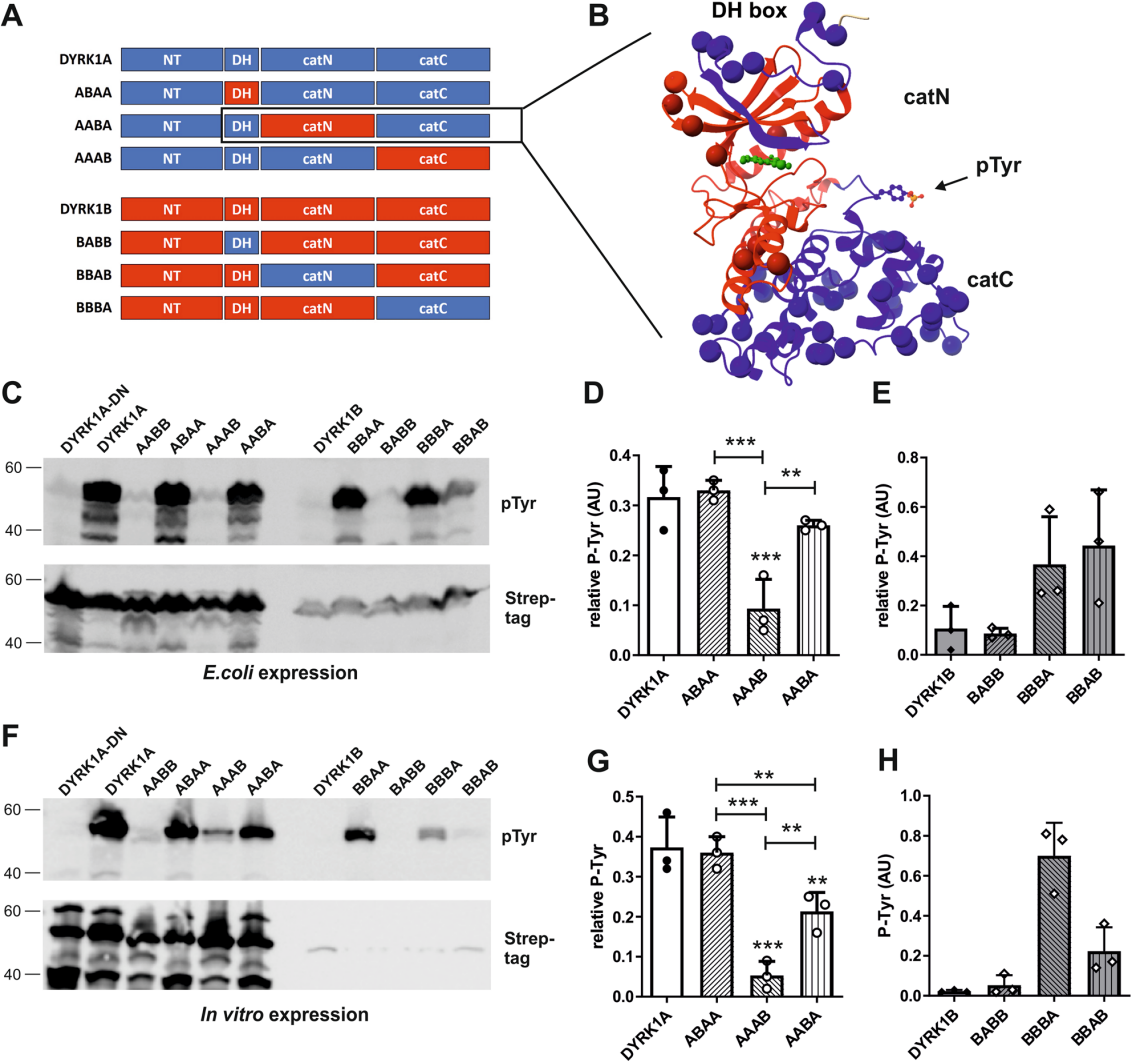
Effects of N-lobe or C-lobe substitution on tyrosine autophosphorylation. (**A**) Schematic representation of the chimeras. The N-terminal and the C-terminal parts of the catalytic domain are designated catN and catC. Information on the amino acid positions of the different parts can be found in the Supplementary information. (**B**) Exemplary representation of the chimeric kinase domain in the AABA construct. The ribbon diagram is based on the structure of a DYRK1A-inhibitor co-crystal (4YLJ^52^). The DH box and catC are shown in blue and the catN part is shown in red. CatN comprises the ATP binding pocket (illustrated by the ball and stick representation of the inhibitor in green) and the catalytic cleft. Spheres indicate the position of amino acids that differ between DYRK1A and DYRK1B. The activation loop with the autophosphorylated tyrosine (pTyr) is identical in DYRK1A and DYRK1B. (**C**–**E**) Bacterial expression. Tyrosine autophosphorylation of the indicated constructs was analyzed by immunoblotting. The graphs show the quantitative results for the chimeric DYRK1A constructs containing segments of DYRK1B (**D**) and the chimeric DYRK1B constructs containing segments of DYRK1A (**E**) (means and SD, n = 3). Quantitation of AABB and BBAA was included in Fig. 4. Unless otherwise indicated, statistical significance is given for the comparison with the wild type constructs. (**F**–**H**) Cell-free expression. The DYRK1A-DYRK1B chimeric constructs were expressed by in vitro translation for 2 h at 37 °C. Panel G shows the relative tyrosine autophosphorylation of the DYRK1A chimeric constructs (means and SD, n = 3). Because of low Strep-tag signal intensities, relative autophosphorylation of DYRK1B constructs could not be calculated. Absolute phosphotyrosine signals are shown in panel H (means and SD, n = 3).

The DH box and the catalytic domain of DYRKs cooperatively form an autonomous folding unit that can be subdivided into three subdomains: the DH box, the N-terminal lobe of the catalytic domain and the C-terminal lobe^21^. To further delineate the structural determinants that distinguish DYRK1A and DYRK1B with respect to chaperone-independent folding, we generated chimeric constructs in which either of these three subdomains of DYRK1A was swapped for the respective region of DYRK1B and vice versa (Fig. 5A). We decided to fuse the N-and C-terminal parts of the catalytic domain in an extended region of sequence identity between DYRK1A and DYRK1B. Therefore, the parts of the chimeric domain do not exactly match with the N-lobe and C-lobes and were termed catN and catC (Fig. 5A,B). Specifically, the D and E helices are included in the catN part (red portion of the C-lobe in Fig. 5B). Owing to the high sequence similarity of DYRK1A and DYRK1B, there are only three diverging amino acids in this region (indicated by spheres). The hinge region and the catalytic cleft that bind ATP and the protein substrate at the interface of the two lobes remain essentially unaltered by the fusion (Fig. 5B).

These chimeras were compared with the wild type kinases to define the roles of the individual subdomains. As shown in Fig. 5, swapping the DH-box between DYRK1A and DYRK1B had no detectable effect on the relative tyrosine autophosphorylation of either kinase (DYRK1A *vs.* ABAA, DYRK1B *vs.* BABB). In contrast, substitution of the C-lobe of DYRK1A by that of DYRK1B (AAAB *vs*. DYRK1A) strongly reduced tyrosine autophosphorylation. Moreover, the reciprocal grafting of the C-lobe of DYRK1A to the N-lobe of DYRK1B supported tyrosine autophosphorylation (BBBA vs. DYRK1B). Exchange of the N-lobes between DYRK1A and DYRK1B had weaker effects. Substitution of the original DYRK1A N-lobe by that of DYRK1B reduced tyrosine phosphorylation in the in vitro translation system (DYRK1A vs. AABA; Fig. 5G) but had little impact in *E. coli* (Fig. 5D). The reciprocal exchange of the N-lobe in DYRK1B appeared to increase autophosphorylation of BBAB vs. DYRK1B (Fig. 5C,E,F,H), although this effect could not be statistically supported. Taken together, the discordant capacity of DYRK1A and DYRK1B to fold in the absence of chaperones is predominately determined by differences in the C-lobe while the DH box had no detectable effect.

### Mammalian expression of DYRK1A-DYRK1B chimeric constructs

Next, we designed mammalian expression constructs analogous to those used for bacterial expression. Constructs contained an N-terminal FLAG tag for immunoprecipitation and a C-terminal HiBiT tag. The HiBiT peptide can be quantitatively detected in cell lysates and on blots using a luciferase-based fragment complementation assay^34^. The HSP90 inhibitor ganetespib was used to assess the chaperone dependence of tyrosine autophosphorylation of wild type and chimeric DYRK1 constructs.

**Figure 6:**
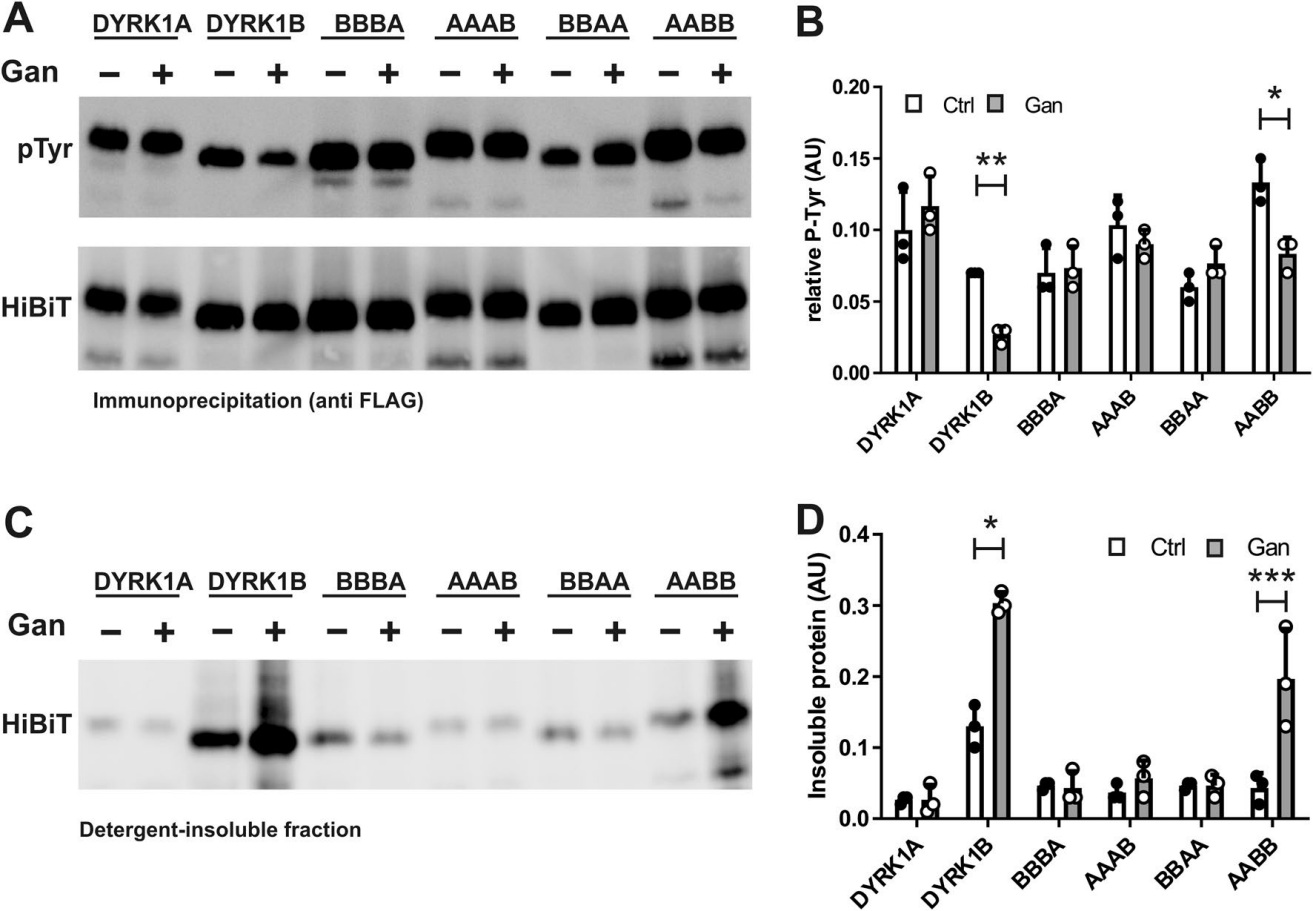
Effect of ganetespib on the maturation of the DYRK1A-DYRK1B chimera in mammalian cells. HEK293 cells transiently expressing DYRK1A, DYRK1B or chimeric constructs with an N-terminal FLAG tag and a C-terminal HiBiT tag were treated with 100 nM ganetespib (Gan) for 24 h or were not treated. Cells were extracted using a non-denaturing lysis buffer, and detergent-soluble fractions and insoluble fractions were separated by centrifugation. (**A**,**B**) Tyrosine autophosphorylation. The relative phosphotyrosine content of DYRK constructs in the soluble fraction was assayed by anti-FLAG immunoprecipitation and immunoblot analysis. HiBiT-containing proteins were detected using a fragment complementation assay (HiBiT blotting detection system). The graph shows means and SD of 3 experiments (**B**). (**C**,**D**) Accumulation in the insoluble fraction. Igepal-insoluble proteins in the pellets were solubilized in SDS buffer and DYRK1 constructs were quantitatively using the HiBiT blotting detection system (**C**). The concentration of HiBiT-tagged proteins in the soluble fraction was measured using the HiBiT lytic detection system. For each construct, the relative proportion of insoluble protein was calculated by dividing the HiBiT signals in the pellet by the HiBiT lytic values for the soluble protein as obtained in the respective supernatants (panel **D**; means and SD, n = 3). Statistical significance is indicated only for the treatment effects (*p < 0.05, **p < 0.01, ***p < 0.001).

In HEK293 cells, relative tyrosine phosphorylation of DYRK1A and DYRK1B did not differ significantly under basal conditions (Fig. 6A,B). This result shows that the impaired autophosphorylation of DYRK1B in *E. coli* was due to the heterologous expression system; and possibly caused by the absence of the CDC37/HSP90 chaperone system. Indeed, inhibition of HSP90 by ganetespib reduced phosphotyrosine levels of DYRK1B and AABB, but not of DYRK1A and BBAA. Thus, the catalytic domain, rather than the N-terminal region or the DH box, accounts for chaperone dependence of DYRK1B during maturation. Interestingly, substitution of the C-lobe in DYRK1B by that of DYRK1A sufficed to render BBBA resistant to the effect of ganetespib. Conversely, grafting the C-lobe from DYRK1B to the catalytic domain of DYRK1A did not sensitize this construct to ganetespib. This result shows that the destabilizing effect of the DYRK1B C-lobe depends on the thermodynamic properties of the entire folding unit and is counterbalanced by the DYRK1A part in the AAAB chimera.

It should be noted that this measurement of phosphotyrosine in immunoprecipitated DYRK1 is likely to underestimate the effect of ganetespib, since unphosphorylated DYRK1B is prone to aggregation and is enriched in the insoluble fraction^16^. Consistent with our previous observation with GFP fusion proteins^16^, ganetespib did not reduce the amount of the soluble DYRK1B in the immunoprecipitates. However, HSP90 inhibition increased the amount of the FLAG-DYRK1B-HiBiT in the insoluble fraction, but not that of the analogous DYRK1A construct (Fig. 6C). This effect could also be attributed to the catalytic domain of DYRK1B (AABB *vs*. BBAA). Again, substitution of the C-lobe by the corresponding sequence of DYRK1A prevented the accumulation of DYRK1B in the insoluble fraction. Unexpectedly, comparison of DYRK1B and AABB also uncovered a role of the N-terminal region, combined with the DH box, on the solubility of the DYRK1B catalytic domain. Substantial amounts of DYRK1B, but not AABB (different at p = 0.0043), were detected in the insoluble fraction of untreated cells (Fig. 6C,D). AABB also contained higher levels of phosphotyrosine than DYRK1B (p = 0.0066). This difference may be due to the fact that the DH box of DYRK1A harbors a phosphorylatable tyrosine (Tyr145)^35^ that is absent in human DYRK1B.

### Thermal stability of DYRK1A-DYRK1B chimeric constructs

**Figure 7:**
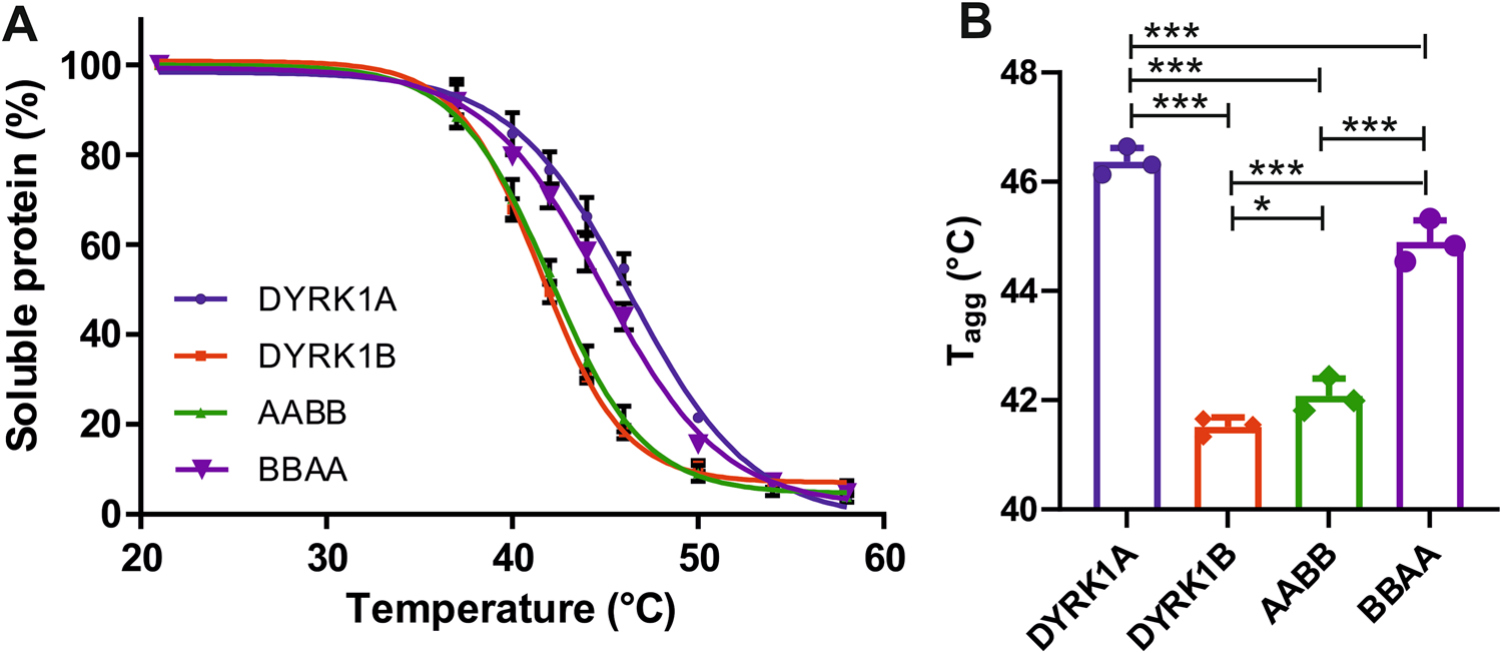
CETSA melt curves of wild type and chimeric DYRK1 constructs. Intact HEK293 cells with stable overexpression of the indicated HiBiT-tagged DYRK constructs were incubated for 3.5 min at variable temperatures. After freeze–thaw lysis, samples were centrifuged and the amount of DYRK constructs in the soluble fraction was measured using the HiBiT lytic assay. The graphs represent the means and SD of 3 independent experiments. (**A**) Curves were fitted to complete data sets for all temperatures, although some symbols and error bars are not shown in the graph. (**B**) The column diagram illustrates the T_agg_ values as determined by curve fitting.

To directly address the question whether DYRK1A and DYRK1B differ in the thermodynamic stability of the mature catalytic domain, we subjected the kinases to cellular thermal shift assays (CETSA)^36^. Suspensions of HEK293 cells expressing wild-type or chimeric FLAG-DYRK1-HiBiT constructs were transiently exposed to increasing temperatures to induce thermal denaturation of the proteins. After cell lysis, HiBiT-tagged proteins in the soluble fractions were quantified using the split luciferase assay. The mid-point of this transition, where 50% of the protein remains in the native and soluble state, is referred to as the aggregation temperature (T_agg_). As shown in Fig. 7, thermal denaturation of DYRK1A was observed at T_agg_ nearly 5 °C higher than T_agg_ of DYRK1B. Analysis of the chimeric constructs AABB and BBAA confirmed that this difference reflects the different conformational stabilities of the catalytic domains.

## Discussion

DYRK1B is a HSP90 client that interacts more strongly with HSP90 and CDC37 than DYRK1A^16,17^. Here we have comparatively characterized DYRK1A and DYRK1B with respect to initial folding as well as the stability of the mature catalytic domain. The present results reveal that DYRK1A and DYRK1B differ strikingly regarding their capacity of autonomous folding. Furthermore, we show that the HSP90 client status of DYRK1B correlates with the thermal instability of its mature kinase domain.

In DYRK kinases, the correct initial folding of the catalytic domain results immediately in the irreversible and stoichiometric autophosphorylation of a tyrosine in the activation loop. This eponymous phenomenon allowed us to monitor correct post-translational folding independent of the later fate of the mature protein^8,9,11^. Bacterial expression in *E. coli* or by in vitro translation showed that the maturation of DYRK1B was severely compromised in the absence of eukaroytic chaperones when directly compared with DYRK1A. Interestingly however, the catalytic domain could fold correctly when a GST-DYRK1B fusion protein was expressed at low temperatures (8 °C), which indicates that the maturation of DYRK1B is not absolutely dependent on HSP90/CDC37 chaperoning. Protein folding is a temperature-dependent process, and our result is in accordance with previous findings that the CDC37 dependence of the yeast protein kinase Sky1 is more stringent at higher growth temperatures^37^. This suggests that the CDC37/HSP90 complex protects the semi-folded nascent kinase chain from aggregation to maintain a folding competent conformation (“holdase” function) but is not required for the actual folding process (“foldase” activity).

Several pairs of closely related kinases with discordant HSP90 client status, such as A-Raf and B-Raf, have been identified in a kinome-wide screen^23^. Evolutionary analysis indicates that the ancestral member of the protein kinase family was not an Hsp90 client, and that kinases are more likely to become HSP90 clients than to lose client status^38^. DYRK1A and DYRK1B originated as paralogous genes early in vertebrate evolution. The sequence of human DYRK1B is by far the most divergent among vertebrate DYRK1A and DYRK1B orthologs (Fig. 3A, supplementary Fig. S1). Interestingly, we found that DYRK1B from zebrafish and from *Xenopus* are well capable of maturation in a cell-free bacterial system, indicating that mammalian DYRK1B became a Hsp90 client long after the duplication of the ancestral DYRK1 gene. The Hsp90 client status has been shown to promote the evolutionary rate of protein kinases, because HSP90 functions as a capacitator of genetic variation by compensating destabilizing mutations^23,39–42^. Thus, the striking sequence divergence of mammalian DYRK1B from its lower vertebrate orthologs may be a consequence of its HSP90 client status.

Owing to their high sequence similarity, DYRK1A and DYRK1B provide a model system for dissecting the structural features that determine the folding properties of a nascent protein kinase domain. Which part of human DYRK1B accounts for its impaired maturation? Mutations in the DH box can impede tyrosine autophosphorylation and compromise the conformational stability of DYRK1A and DYRK1B^16,21^. Moreover, the DH box of human DYRK1B harbors several amino acids that are not found in the other vertebrate class 1 DYRKs (supplementary Fig. S1). However, the reciprocal substitution of the DH box in the DYRK1A/DYRK1B chimera excluded the possibility that these differences account for the differential autophosphorylation of DYRK1A and DYRK1B. In contrast, the capacity of folding without chaperones in the bacterial expression systems was determined by the catalytic domain itself. This conclusion was supported by the analysis of chimeric DYRK1A/DYRK1B constructs in mammalian cells. HSP90 inhibition reduced tyrosine phosphorylation and induced aggregation of DYRK1B and the AABB chimera but did not affect DYRK1A.

The observation that DYRK1B accumulates in the insoluble fraction is consistent with previous results from Taipale et al.^23^ who distinguished HSP90 client kinases that either are degraded upon HSP90 inhibition or form intracellular aggregates. In their kinome screen, as well as in our previous analysis of GFP-DYRK1B constructs^16^, cellular DYRK1B levels were not reduced in ganetespib treated cells. In contrast, Miyata et al.^17^ reported reduced levels of DYRK1B in the soluble fraction of geldanamycin-treated COS7 cells (Fig. 4). In our previous study^16^, total cellular levels of GFP-DYRK1B in HeLa cells were not detectably changed by ganetespib (Fig. 3A). Differences in the level of overexpression, the epitope tags used or the cellular systems may have affected the balance between degradation and aggregation of DYRK1B in these studies.

Our further analysis of the constructs with chimeric kinase domains identified the C-lobe as the main structural element that distinguishes DYRK1A and DYRK1B regarding their maturation in bacterial expression systems. This was an unexpected result, because the deviant residues in the C-lobe of DYRK1B are rather distant from the catalytic cleft and the autophosphorylation site in the activation loop (Fig. 5B). Interestingly, hydroxylation of a conserved proline residue (Pro380 in DYRK1A, Pro332 in DYRK1B) in the C-lobe has recently been reported to be essential for tyrosine autophosphorylation of DYRK1A and DYRK1B^43^. This proline is located at the N-terminal end of the CMGC insert (also known as MAPK insert), a segment characteristic for members of the CMGC group of protein kinases^44^. The critical proline is conserved, but the CMGC insert is the most variable sequence in the catalytic domain of class 1 DYRKs (supplementary Fig. S1). It appears plausible that these differences determine the differential maturation in DYRK1A and DYRK1B. This hypothesis implies that the CMGC insert has a critical function independent of proline hydroxylation, since this modification cannot take place in the bacterial expression systems.

Given that client recognition by HSP90 is determined by thermodynamic parameters, the autophosphorylated mature catalytic domain of DYRK1B is expected to be conformationally less stable than that of DYRK1A. Indeed, we found that bacterially expressed DYRK1B was much more sensitive to thermal inactivation than DYRK1A. In mammalian cells, thermal denaturation of DYRK1B in CETSA experiments was observed at significantly lower temperatures than that of DYRK1A. This low thermal stability of DYRK1B may be the reason why its crystal structure has so far not been solved. Nevertheless, a hypothetical structure of DYRK1B can be modeled based on available DYRK1A structures without obvious structural perturbations^16,18^. The residues diverging between DYRK1A and DYRK1B are exposed to the surface of the protein and have no apparent role in the overall domain fold. All amino acids that contribute to the regulatory (R) and catalytic (C) hydrophobic “spines” that define the core architecture of the active conformation of protein kinases are invariable between DYRK1A and DYRK1B^45^. In addition, the residues (Glu331 and Arg467 in DYRK1A) forming the conserved salt bridge that is buried in the C-lobe of the protein kinase domain^46^ are also conserved in DYRK1B. In a previous study, Miyata and Nishida replaced several single residues in DYRK1A with the corresponding amino acid of DYRK1B but observed no effect on chaperone binding^17^. Further work will be necessary to precisely define the structural differences between DYRK1A and DYRK1B that control both the initial folding of the nascent protein and the conformational stability of the mature catalytic domain.

## Materials and methods

### Antibodies

The following antibodies were used for the detection of phosphotyrosine levels of the DYRK1 constructs: rabbit polyclonal antibodies raised against phosphotyrosine in the activation loop of DYRK1A/DYRK1B (1:1000, Phospho-DYRK1A/DYRK1B pTyr321, pTyr273, Thermo Fisher Scientific #PA5*-*64574; RRID:AB_2663607) or against phospho-HIPK2 (1:500 Phospho-HIPK2 pTyr361, Thermo Fisher Scientific #PA5-13045, RRID:AB_10987115) and a mouse monoclonal antibody against phosphorylated tyrosines (1:1000 pY99, #sc-7202 Santa Cruz Biotechnology #sc-7020, RRID:AB_628123). Polyclonal goat anti GST antibody (1:1000) was taken from the GST Detection Module (GE healthcare, 27-4590). Horseradish peroxidase-coupled secondary antibodies were as follows: donkey polyclonal anti-rabbit IgG (H&L, #611-7302, Rockland, Gilbertsville, PA, USA), donkey polyclonal anti-mouse IgG (H&L, #610-703-124, Rockland, Gilbertsville, PA, USA), goat anti-biotin IgG (#7075, Cell Signaling Technology, Danvers, MA, USA).

### Plasmids

The NEBuilder Assembly Tool v2.2.7 and the NEBuilder HiFi DNA Assembly Cloning Kit (#E5520S, New England Biolabs, Ipswich, MA, USA) were used to plan and generate bacterial and mammalian expression plasmids, to construct chimeric cDNAs, and to introduce FLAG and HiBiT tags. The QuikChange method was employed for site-directed mutagenesis. For bacterial expression and in vitro translation, cDNA encoding the conserved regions (ΔC) of class 1 DYRKs were inserted between the NheI and EcoRI sites of the expression vector pET-ST2^11^ to create an N-terminal fusion with the Strep-tag 2 sequence. The pGEX-2TK vector was used for bacterial expression of C-terminally deleted GST-DYRK1 fusion proteins. The plasmid vector pcDNA5/FRT/TO (#V652020, Thermo Fisher Scientific, Rockford, IL, USA) was used for transient and stable mammalian expression of DYRK1A-DYRK1B constructs with an N-terminal FLAG tag and C-terminal HiBiT tag. All human DYRK1B cDNAs in this study are derived from the catalytically active DYRK1B-p69 splicing variant^47^. A detailed description of the expression constructs is provided in the supplementary information.

### In vitro translation

Cell free expression of the DYRK1 constructs was performed with the PURExpress In Vitro Protein Synthesis Kit (#E6800S, New England Biolabs, Ipswich, MA, USA). PURExpress is a coupled in vitro-transcription/translation system reconstituted from recombinant purified components necessary for *E. coli* translation. Reactions were incubated for 2 h at 37 °C according to the manufacturer’s instructions but were downscaled and contained either 100 ng plasmid DNA and 1 µL murine RNase-Inhibitor (#M0314S, New England Biolabs, Ipswich, MA, USA) in a total volume of 10 µl (Fig. 3B) or 45 ng plasmid DNA and 0.3 µl RNase-Inhibitor in a total volume of 6 µL (Figs. 4F–H, 5F–H). Reactions were stopped by adding Laemmli’s sample buffer supplemented with DTT (200 mM) and incubated for 10 min at 96 °C before samples were subjected to SDS-PAGE and Western blot analysis.

### Bacterial expression

The pET expression vectors were transformed into competent *E. coli* BL21 (DE3) (#C2527I, New England Biolabs, Ipswich, MA, USA). Single colonies were used for inoculation of a starter culture of 1 mL LB medium containing 30 µg/mL kanamycin. Starter cultures were incubated overnight at 37 °C, 225 rpm and then diluted 1:20 (50 µL) into 1 mL selective LB medium. These cultures were incubated for 3 h at 37 °C at 225 rpm before protein expression was induced with isopropyl thiogalactoside (IPTG, final concentration 0.1 mM). Expression proceeded for 4 h at 37 °C, 225 rpm. The cells were harvested by centrifugation. Cell pellets were lysed with 80 µL 1 × Laemmli’s sample buffer containing 200 mM DTT for 10 min at 96 °C to prepare total cell lysates for Western blot analysis.

GST fusion proteins were expressed in *E. coli* BL21 Rosetta cells (Novagen). Cells were grown in LB medium containing 100 µg/mL ampicillin at 37 °C to an OD_600_ of 0.7 and distributed in test tubes (4 aliquots of 3 ml). Expression was induced by adding IPTG to a final concentration 0.1 mM and proceeded for either 4 h at 37 °C or 23 h at 19 °C under orbital shaking or for 48 h at 8 °C (tumble shaker). Cells were lysed by sonication under native conditions (phosphate buffered saline, 1% triton X-100) at 0–4 °C, lysates were cleared by centrifugation and supernatants were incubated with glutathione sepharose CL-4B (10 µL bed volume per sample) for 2 h at 4 °C. The affinity matrix was washed twice with 1 ml PBS before bound proteins were eluted. For immunoblot analysis, bound GST fusion proteins were eluted under denaturing conditions (Laemmli’s sample buffer, 200 mM DTT, 96 °C) and the amounts of samples loaded on the gels were adjusted according to Coomassie staining intensities. For assays of catalytic activity, GST-DYRK1A (expressed at 37 °C) and GST-DYRK1B (expressed at 8 °C) were eluted under native conditions using 50 mM Tris–HCl pH 8.0, 10 mM reduced glutathione and stored at − 80 °C until use.

### Kinase assays after thermal challenge

GST-DYRK1 fusion proteins were aliquoted at amounts that resulted in the depletion of approximately 90% of the ATP in the Kinase-GLO Luminescent Kinase Assay from Promega (linear range of the kinase titration curve). These aliquots were incubated for 3.5 min at temperatures between 39 °C and 47 °C as indicated in Figs. 2C and S2. Immediately thereafter, assays were run in a total volume of 15 μL with kinase-buffer (25 mM Hepes pH 7.4, 0.5 mM DTT, 5 mM MgCl_2_) in the presence of 5 µM ATP and 20 μM DYRKtide as a substrate peptide. Reactions proceeded at room temperature for 30 min before the luciferase reaction was run to determine the ATP depletion by the kinase reaction. The decrease in ATP levels after the reaction was used as a measure of catalytic kinase activity.

### Cell culture and transfection

HEK293 cells were expanded from cryopreserved stocks retained from previous studies. Cells were grown in Dulbecco’s modified Eagle medium/F-12 (DMEM/F-12, #11330057, Thermo Fisher Scientific, Rockford, IL, USA) with 10% fetal bovine serum and kept at 37 °C in a humidified 5% CO_2_ atmosphere. For immunoprecipitation experiments, HEK293 cells were seeded into 100-mm dishes (730.000 cells per 100-mm plate) and transiently transfected using the FuGENE HD Transfection Reagent (#E2311, Promega Corporation, Madison, WI, USA) on the following day. Stable isogenic Flp-In T-Rex 293 cell lines for doxycycline-inducible expression of FLAG-DYRK1-HiBiT variants were established by Flp recombinase-mediated integration of the pcDNA5/FRT/TO expression vector according to the manufacturer’s instructions (Invitrogen, Carlsbad, Ca, USA). The 293 parental cell line was from ATCC (#CRL-1573).

### Immunoprecipitation of FLAG tag fusion proteins

HEK293 cells transiently expressing the FLAG- and HiBiT-tagged DYRK1 constructs were treated with either 100 nM ganetespib (#9459, BioVision Incorporated, Milpitas, CA, USA) or vehicle (DMSO). Twenty-four hours later, cells were washed with PBS at room temperature and then lysed in non-denaturing immunoprecipitation buffer (700 µL/10-cm plate; 50 mM Tris pH 7.4, 150 mM NaCl, 0.5% Igepal CA 630, 15% glycerol, 1 mM EDTA, 1 mM NaF) supplemented with phosphatase- and protease inhibitors (1 mM Na_3_VO_4_, 1 mM PMSF, 10 µg/mL each of pepstatin and aprotinin, and 12.2 µg/mL leupeptin). After dishes were shaken on ice for 20 min, lysates were vortexed and cleared by centrifugation (10 min, 14,000 rpm, 4 °C). Cell pellets were stored at − 20 °C for blotting analysis of HiBiT-tagged proteins in the insoluble fraction. Aliquots of 10 µL of the supernatants were used for Nano-Glo HiBiT lytic assay (#N3030, Promega Corporation, Madison, WI, USA) to determine the concentration of HiBiT-tagged proteins in the soluble fraction. For immunoprecipitation, 660 µL of each supernatant was incubated overnight at 4 °C with 40 µL of anti-FLAG M2 affinity gel (mouse monoclonal antibody covalently coupled to agarose beads; #A4220, Sigma-Aldrich, St. Louis, MO, USA). The beads were washed three times with an excess of washing buffer (50 mM Tris pH 7.4, 150 mM NaCl, 1 mM EDTA) and collected by centrifugation (30 s, 7000 g, 4 °C). Aliquots of the lysate were taken before and after immunoprecipitation to confirm the efficiency of the immunoprecipitation with the help of the HiBiT lytic assay (routine recovery about 80%). Bound proteins were eluted in 30 µL 1 × Laemmli’s sample buffer without DTT at 96 °C for 10 min, and 15 µL were then subjected to SDS-PAGE. DTT was omitted to avoid the co-migration of the IgG heavy chain (liberated from the FLAG affinity resin) with the FLAG-DYRK1-HiBiT constructs, which severely impaired the detection of pTyr.

### Western blotting

Protein samples were separated by SDS-PAGE in 10% acrylamide gels and blotted onto nitrocellulose membranes (#1060002, Amersham Protran 0.45 μm NC, GE Healthcare Life Sciences, Marlborough, MA, USA). Membranes were blocked with 3% bovine serum albumine in TBS-T buffer (50 mM Tris pH 7.5, 150 mM NaCl, 0.1% Tween-20). Recombinant proteins were detected using the Strep-Tactin horseradish peroxidase HRP) conjugate (#2-1502-001, IBA, Göttingen, Germany) or the Nano-Glo HiBiT blotting system (#N2410, Promega, Madison, WI, USA) according to the manufacturers’ instructions. To determine anti-phosphotyrosine immunoreactivity, membranes were probed with primary antibodies overnight at 4 °C, followed by incubation with HRP-coupled secondary antibodies (Rockland) for 1 h at room temperature. Chemiluminescence signals were detected with the help of LAS-3000 imaging system (Fujifilm, Düsseldorf, Germany) and band intensities were quantified using AIDA image analysis software (Raytest, Straubenhardt, Germany) or Multi Gauge Analysis Software (Fujifilm) for Fig. 6. Relative phosphotyrosine content was calculated as the ratio of p-Tyr to the Strep-tag or HiBiT band intensities. For the quantitative analysis of replicate experiments, values (i.e. expression level or relative phosphotyrosine content) for each sample were normalized to the sum of all relevant data points from one blot^48^. Expression constructs with Strep-tag signal intensities ten times lower than wild-type DYRK1A/DYRK1B (Figs. 4, 5) were excluded from statistical analysis. Original uncropped images of the blots are provided as supplementary information.

### Quantification of native and insoluble HiBiT-tagged protein in HEK cells

Detergent-insoluble pellets and the corresponding supernatants from immunoprecipitation experiments were used for relative quantification of soluble and aggregated DYRK1 constructs. HiBiT-tagged proteins in the soluble fraction were measured with the help of Nano-Glo HiBiT lytic assay (see above). The pellets were dissolved in 93 µL 0.5 × Laemmli’s sample buffer without DTT. Samples were incubated for 5 min at 96 °C, followed by vortexing (15 s), sonication, vortexing, and a second incubation step for 10 min at 96 °C. Solubilized proteins (20 µL) were then separated by SDS-PAGE and transferred onto nitrocellulose membranes for detection by the Nano-Glo HiBiT blotting system (#N2410, Promega Corporation, Madison, WI, USA). The relative proportion of insoluble protein was calculated by dividing HiBiT signals on the membranes by the HiBiT values in the corresponding supernatants.

### Cellular thermal shift assay (CETSA)

Stable generated cell lines were seeded on tissue culture dishes 100, (Cell + , 83.3902.300, Sarstedt, Nümbrecht, Germany) at 4 × 10^5^ cells/mL and harvested 48 h after induction with doxycycline by trypsinisation. Detached cells were centrifuged (10 min, 150 g, 4 °C), pelleted cells resuspended in 1 mL ice-cold PBS including a protease inhibitor cocktail (10 μg/mL aprotinin and pepstatin A, 16 μg/μL leupeptin, 1 mM PMSF) and aliquoted to 0.2 mL soft PCR tubes at 100 µL. Samples were heated for 3.5 min at ascending temperatures (RT, 37 °C,40 °C, 42 °C, 44 °C, 46 °C, 50 °C, 54 °C, 58 °C) using a thermal cycler (UNO II, Biometra, Göttingen, Germany) and then equilibrated to room temperature for 3 min. Cells were lysed by three consecutive freeze–thaw cycles between liquid nitrogen and a water bath at room temperature. HiBiT-tagged DYRK1 construct in the supernatant were measured using the HiBiT lytic assay.

### Construction of the dendrogram, molecular visualization and statistics

The maximum likelihood tree was conducted using the MEGA X software^49^ after ClustalW alignment and default settings (for accession numbers see supplementary table S1). The dendrogram was designed with the FigTree Software 1.4.4 (http://tree.bio.ed.ac.uk/software/figtree/) and iTOL v4^50^. The DYRK1 kinase domain was visualized using UCSF ChimeraX 1.0 (Resource for Biocomputing, Visualization, and Informatics at the University of California, San Francisco, with support from NIH R01-GM129325 and the Office of Cyber Infrastructure and Computational Biology, National Institute of Allergy and Infectious Diseases)^51^. Data were statistically evaluated by Generalized Linear Mixed Model (GLMM) Analysis (SAS 9.4, SAS Institute, Cary, NC, USA) except Fig. 1D which was analyzed by one sample t-test (GraphPad PRISM 5.0, GraphPad Software, La Jolla, CA, USA). P-values < 0.05 were considered as significant: *p < 0.05, **p < 0.01, ***p < 0.001. Details of the structural visualization and statistical methods are provided in the supplementary information.

## Supplementary Information

Supplementary Information.

## Abbreviations

CETSA: Cellular thermal shift assay
DH box: DYRK homology box
DTT: Dithiothreitol
GST: Glutathione S-transferase
