# Supplementary material for: Differential maturation and chaperone dependence of the paralogous protein kinases DYRK1A and DYRK1B
Source: Sci Rep. 2022 Feb 14;12:2393. doi: 10.1038/s41598-022-06423-0 (PMC8844047; doi:10.1038/s41598-022-06423-0)

Papenfuss M, Lützow S, Wilms G, Babendreyer A, Flaßhoff M, Kunick C, Becker W

Differential maturation and chaperone dependence of the paralogous protein kinases DYRK1A and DYRK1B

Supplementary Material

1. Construction of expression plasmids

<u>pET-ST2 constructs</u>

The pET28a-derived expression vector for T7 polymerase-driven expression *in vitro* and in *E. coli* BL21(DE3) was described previously (Walte et al. 2013, supplementary material; Fig. S1). The (ΔNΔC) constructs of rDYRK1A and hDYRK1B have also been described before and were designated DYRK1Acat and DYRK1Bcat in this report (Walte et al. 2013). The NEBuilder Assembly Tool v2.2.7 and the NEBuilder HiFi DNA Assembly Cloning Kit (#E5520S, New England Biolabs, Ipswich, MA, USA) were used to plan and generate the new ΔC expression plasmids and to construct chimeric DYRK1A-DYRK1B cDNAs. The pET-ST2 plasmids was linearized by restriction digestion with *Nhe*I and *Eco*RI, and cDNA inserts were PCR amplified with Q5 High-Fidelity DNA Polymerase (#M0491S, New England Biolabs, Inc., Ipswich, MA, USA). Details of the cloned cDNA inserts are listed in Table S1. All cDNAs were completely sequenced to verify the fidelity of the PCR.

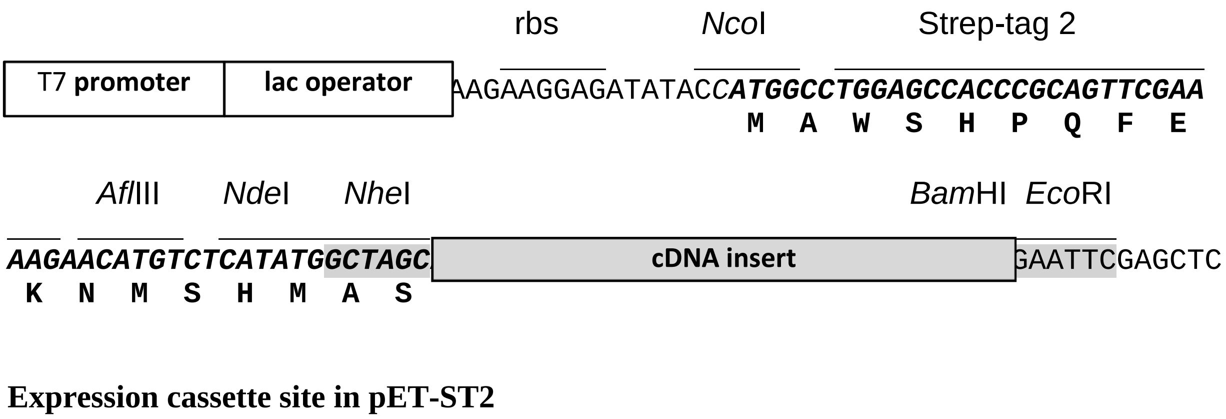

Expression cassette site in pET-ST2
cDNAs were inserted between the *Nhe*I and *Eco*RI sites and contain a stop codon before the *Eco*RI site. rbs, ribosomal binding site

pET-ST2 expression clones for T7-dependent expression of class I DYRKs

ST2 fusion protein	Species	NCBI Refseq accession number[a]	amino acids[b]	Source of template
rDYRK1A-**ΔNΔC**	*Rattus norvegicus*	NP_036923.1	135-499	Kentrup et al. 1996
rDYRK1A-**ΔC**	*Rattus norvegicus*	NP_036923.1	28-499	Kentrup et al. 1996
hDYRK1B-**ΔNΔC**	*Homo sapiens*	NP_004705.1	87-454	Leder et al. 1999
hDYRK1B-**ΔC**	*Homo sapiens*	NP_004705.1	1-454	Leder et al. 1999
xDYRK1B-**ΔC**	*Xenopus laevis*	NP_001080262	1-451	Lilienthal et al. 2010
zDYRK1A-**ΔC**	*Danio rerio*	NP_001074158	47-503	Buchberger et al 2021
zDYRK1B-**ΔC**	*Danio rerio*	XP_005158272.1	1-458	Nissen et al. 2006
MNB-**ΔC**	*Drosophila melanogaster*	NP_728104	66-505	Becker et al. 1998

[a] http://www.ncbi.nlm.nih.gov/protein/

[b] A stop codon was introduced immediately after the last amino acid by design of the reverse PCR primer.

Definition of subdomains for construction of the rDYRK1A-hDYRK1B chimera

Designation of subdomain	Rat DYRK1A (NP_036923.1)	Human DYRK1B (NP_004705.1)	Relevant features
NT	28-135	4-87	DCAF7 binding site
DH	136-170	88-122	Phosphorylated Y145 in DYRK1A
catN	171-316	123-268	ATP binding site, hinge region
overlap[a]	270-335	222-287	*Activation loop, substrate binding site*
catC	317-499	269-454	CMGC insert

[a] region of 100% sequence identity in DYRK1A and DYRK1B. This sequence is identical in all chimera.

pGEX-2TK constructs

The C-terminally deleted GST-rDYRK1AΔC construct (containing amino acids 1-499 of rat DYRK1A) was described earlier (Himpel et al. 2000). To construct GST-hDYRK1BΔC, the cDNA insert of pET-ST2-hDYRK1B (see above) was transferred between the BamHI and EcoRI sites of to pGEX-2TK (Acc. No. U13851) using the NEBuilder HiFi DNA Assembly Cloning Kit.

References for the cDNA clones used as templates:

Becker W, Weber Y, Wetzel K, Eirmbter K, Tejedor FJ, Joost HG. Sequence characteristics, subcellular localization, and substrate specificity of DYRK-related kinases, a novel family of dual specificity protein kinases. *J Biol Chem* 273:25893-902 (1998).

Buchberger A, Schepergerdes L, Flaßhoff M, Kunick C, Köster RW. A novel inhibitor rescues cerebellar defects in a zebrafish model of Down syndrome-associated kinase Dyrk1A overexpression. *J Biol Chem* 297:100853 (2021).

Kentrup H, Becker W, Heukelbach J, Wilmes A, Schürmann A, Huppertz C, Kainulainen H, Joost HG Dyrk, a dual specificity protein kinase with unique structural features whose activity is dependent on tyrosine residues between subdomains VII and VIII. *J Biol Chem* 271:3488-95 (1996).

Leder S, Weber Y, Altafaj X, Estivill X, Joost HG, Becker W. Cloning and characterization of DYRK1B, a novel member of the DYRK family of protein kinases. *Biochem Biophys Res Commun* 254:474-9 (1999).

Lilienthal E, Kolanowski K, Becker W. Development of a sensitive non-radioactive protein kinase assay and its application for detecting DYRK activity in Xenopus laevis oocytes. *BMC Biochem* 11:20 (2010).
Nissen RM, Amsterdam A, Hopkins N. A zebrafish screen for craniofacial mutants identifies wdr68 as a highly conserved gene required for endothelin-1 expression. *BMC Dev Biol* 6:28 (2006).

Mammalian expression constructs

The plasmid vector pcDNA/FRT/TO was equipped with an N-terminal FLAG tag to allow standardized immunoprecipitation of different recombinant proteins and a C-terminal HiBiT tag for antibody-free quantification of protein levels (see below). The cDNA inserts were transferred from the respective pET-ST2 plasmids to the pcDNA/FRT/TO-FLAG-HiBiT vector with the help of the NEBuilder Kit.

```
                                                HindIII
GATCCAGCCTCCGGACTCTAGCGTTTAAACTTAAGCTTCGTTTAGTGACCGTCAGAATTGATCTA

 NcoI                                       NdeI    EcoRV   BamHI
CCATGGACTACAAAGACGATGACGACAAGCATATGTCCGATATCGGATCCGTGAGCGGCTGGCGG
   M  D  Y  K  D  D  D  D  K  H  M        G  S  V  S  G  W  R

                                    NotI
CTGTTCAAGAAGATTAGCTAGGCGGCCGCTCGAGTCTAGAGGGCCCGTTTAAACCCGCTGATCAGCCT
 L  F  K  K  I  S  *
```

Cloning site of pcDNA/FRT/TO-FLAG-HiBiT
The sequence inserted between the HindIII and NotI sites of the original pcDNA/FRT/TO (#V652020, Thermo Fisher Scientific, Rockford, IL, USA) is shown in italics. Sequences coding for the FLAG tag (blue) and the HiBiT tag (red) are highlighted. The boxed sequence with the EcoRV site was eliminated when cDNAs were inserted by Gibson cloning.

2. Methods and software for molecular visualization and statistical evaluation

Molecular visualization

The structure of the chimeric kinase domain was visualized using UCSF ChimeraX 1.0 (Resource for Biocomputing, Visualization, and Informatics at the University of California, San Francisco, with support from NIH R01-GM129325 and the Office of Cyber Infrastructure and Computational Biology, National Institute of Allergy and Infectious Diseases) [Kumar et al. 2018]. Water and ethylene glycol molecules and sulfate ions in the DYRK1A (4YLK) [Letunic et al. 2019] were hidden using UCSF ChimeraX. Alpha C atoms of non-matching amino acids from the sequence alignment were visualized as spheres. Subdomains of the chimeric construct (A)ABA were colored according to their origin: catN (amino acids 171 – 316) in red, with non-matching amino acids shown as dark red spheres; DH box (136 – 170) and catC (317 – 481) in blue, with non-matching amino acids shown as dark blue spheres. The first part of the chimeric construct (NT of DYRK1A) is not present in the crystal structure.

Codon usage analysis

The coding sequences in the pET-ST2-DYRK1A-ΔC and pET-ST2-DYRK1B-ΔC constructs were analyzed for the fraction of rare codons using the E. coli Codon Usage Analyzer 2.1 (http://www.faculty.ucr.edu/~mmaduro/codonusage/usage.htm). The codon adaptation index (Sharp and Li 1987) was used as a global measure of the resemblance between the synonymous codon usage of the expression constructs with the codon frequencies of *E. coli*.

Statistical methods

Data values were calculated from at least three independent experiments and are shown as means and standard deviation (SD). All data were analyzed by Generalized Linear Mixed Model (GLMM) Analysis (SAS 9.4, SAS Institute, Cary, NC, USA) except Fig. 1D which was analyzed by one sample t-test (GraphPad PRISM 5.0, GraphPad Software, Inc., La Jolla, CA, USA). GLMM analysis was applied to find out differences between included DYRK1 constructs regarding expression, relative catalytic activity and conformational stability under ganetespib treatment. Distribution of the residuals was analyzed using residual plots and the Shapiro-Wilk test. If necessary, the experiment was set as random term to assess for experiment-specific differences. In the case of heteroscedasticity (according to the covtest statement) the degrees of freedom were adjusted by the Kenward-Roger approximation. In terms of multiple comparisons, p-values were corrected by false discovery rate (FDR). Diagrams were created using GraphPad PRISM 5.0 (GraphPad Software, La Jolla, CA, USA).

References

Kumar S, Stecher G, Li M, Knyaz C, Tamura K. MEGA X: Molecular Evolutionary Genetics Analysis across computing platforms. *Mol Biol Evolution* **35**, 1547-1549 (2018).

Letunic I, Bork P. Interactive Tree Of Life (iTOL) v4: recent updates and new developments. *Nucleic Acids Res* **47**, W256-W259 (2019).

Sharp PM, Li WH. The codon Adaptation Index--a measure of directional synonymous codon usage bias, and its potential applications. *Nucleic Acids Res*. **15**, 1281-95 (1987)

3. Supplementary Figures and Tables:

```
rDyrk1A     30  MAAQMPHSHQ-YSDRRQPNISDQQVSALSYSDQIQQPLTNQVMPDIVMLQRRMPQTFRDP
zDYRK1Aa    34  MDAQTPHSHPPFSDAHPQGAADQSAAALPYNPQAPQPSATQVTADMVMLQRRMPQCFRDP
hDYRK1B      1  M--AVPPGHGPFSGFPG--PQ----------------EHTQVLPDVRLLPRRLPLAFRDA
xDYRK1B      1  M--SSQHSHAPFSSLQS--MA----------------DHQQGLPDMTILQRRIPLTFRES
zDYRK1B      1  M--SSQHSHPSFSNIHS--MA----------------DQQQVLSDMTILQRRIPPSFRDP
 MNB        66  M--HH-HSSP------------------------------SSSSEVRAMQARIPNHFREP

                     DCAF7-binding site       NLS                NLS  |ooo-DH box-
rDyrk1A     89  ATAPLRKLSVDLIKTYKHINEVYYAKKKRRHQQGQGD-DSSHKKERKVYNDGYDDDNYDY
zDYRK1Aa    94  ATAPLRKLSIELIKTYKHINEVYYAKKKRRHQQGQGE-DSSNKKERKIYNDGYDDDNFDY
hDYRK1B     41  TSAPLRKLSVDLIKTYKHINEVYYAKKKRRAQQAPPQ-DSSNKKEKKVLNHGYDDDNHDY
xDYRK1B     41  AAAPLRKLSVDLIKTYKHINEVYYTKKKRRAQQVPPE-DSSTKKERKVFNDGFDDDNYDY
zDYRK1B     41  ASAPLRKLSVDLIKTYKHINEVYYTKKKRRAQQVPPE-DSSTKKERKVYNDGYDDDNYDY
 MNB        93  ASGPLRKLSVDLIKTYKHINEVYYAKKKRRAQQTQGDDDSSNKKERKLYNDGYDDDNHDY

                oooooo      |--- catalytic domain : N lobe -----------------------------
rDyrk1A    148  IVKNGEKWMDRYEIDSLIGKGSFGQVVKAYDRVEQEWVAIKIIKNKKAFLNQAQIEVRLL
zDYRK1Aa   153  IVKNGEKWMDRYEIDSLIGKGSFGQVVKAYDRVEQEWVAIKIIKNKKAFLNQAQIEVRLL
hDYRK1B    100  IVRSGERWLERYEIDSLIGKGSFGQVVKAYDHQTQELVAIKIIKNKKAFLNQAQIELRLL
xDYRK1B    100  IVKNGEKWMDRYEIDSLIGKGSFGQVVKAYDHHDQEWVAIKIIKNKKAFLNQAQIELRLL
zDYRK1B    100  IVKNGEKWLDRYEIDSLIGKGSFGQVVKAYDHHEQEWVAIKIIKNKKAFLNQAQIELRLL
 MNB       153  IIKNGEKFLDRYEIDSLIGKGSFGQVVKAYDHEEQCHVAIKIIKNKKPFLNQAQIEVKLL

                ---------------- N lobe ------HINGE-----C lobe ----------------
rDyrk1A    208  ELMNKHDTEMKYYIVHLKRHFMFRNHLCLVFEMLSYNLYDLLRNTNFRGVSLNLTRKFAQ
zDYRK1Aa   213  ELMNKHDTEMKYYIVHLKRHFMFRNHLCLVFEMLSYNLYDLLRNTNFRGVSLNLTRKFAQ
hDYRK1B    160  ELMNQHDTEMKYYIVHLKRHFMFRNHLCLVFELLSYNLYDLLRNTHFRGVSLNLTRKLAQ
xDYRK1B    160  ELMNKHDTEMKYYIVHLKRHFMFRNHLCLVFELLSYNLYDLLRNTNFRGVSLNLTRKFAQ
zDYRK1B    160  ELMNKHDTEMKYYIVHLKRHFMFRNHLCLVFELLSYNLYDLLRNTNFRGVSLNLTRKFAQ
 MNB       213  EMMNRADAENKYYIVKLKRHFMWRNHLCLVFELLSYNLYDLLRNTNFRGVSLNLTRKFAQ

                -------------------C lobe---------------- ••• activation segment •••
rDyrk1A    268  QMCTALLFLATPELSIIHCDLKPENILLCNPKRSAIKIVDFGSSCQLGQRIYQYIQSRFY
zDYRK1Aa   273  QLCTALLFLATPELSIIHCDLKPENILLCNPKRSAIKIVDFGSSCQLGQRIYQYIQSRFY
hDYRK1B    220  QLCTALLFLATPELSIIHCDLKPENILLCNPKRSAIKIVDFGSSCQLGQRIYQYIQSRFY
xDYRK1B    220  QLCTALLFLATPELSIIHCDLKPENILLCNPKRSAIKIVDFGSSCQLGQRIYQYIQSRFY
zDYRK1B    220  QLCTALLFLATPELSIIHCDLKPENILLCNPKRSAIKIVDFGSSCQLGQRIYQYIQSRFY
 MNB       273  QLCTALLFLSTPELNIIHCDLKPENILLCNPKRSAIKIVDFGSSCQLGQRIYHYIQSRFY

                •••••--------------- C lobe --------------------------- >>>>>>>>>>--
rDyrk1A    328  RSPEVLLGMPYDLAIDMWSLGCILVEMHTGEPLFSGANEVDQMNKIVEVLGIPPAHILDQ
zDYRK1Aa   333  RSPEVLLGMPYDLAIDMWSLGCILVEMHTGEPLFSGANEVDQMNKIVEVLGVPPSHILEQ
hDYRK1B    280  RSPEVLLGTPYDLAIDMWSLGCILVEMHTGEPLFSGSNEVDQMNRIVEVLGIPPAAMLDQ
xDYRK1B    280  RSPEVLLGMPYDLAIDMWSLGCILVEMHTGEPLFSGSNEVDQMNKIVEVLGTPPNHMLDQ
zDYRK1B    280  RSPEVLLGMPYDLAIDMWSLGCILVEMHTGEPLFSGSNEVDQMNKIVEVLGVPPNHMLDQ
 MNB       333  RSPEVLLGIQYDLAIDMWSLGCILVEMHTGEPLFSGCNEVDQMNKIVEVLGMPPKYLLDQ

                >>>>>>>>>>>>>>>>-CMGC insert-<<<<<<<<<<<<<<<<<<<<<<<<<<<<<<<<
rDyrk1A    388  APKARKFFEKL-PDGTWSLKKTKDGKREYKPPGTRKLHNILGVETGGPGGRRAGESGHTV
zDYRK1Aa   393  APKARKFFEKM-PDSMWCAKKTKDGKR-YKPAGSRKLHSILGVEAGGPGGRRAGESGHAV
hDYRK1B    340  APKARKYFERL-PGGGWTLRRTKELRKDYQGPGTRRLQEVLGVQTGGPGGRRAGEPGHSP
xDYRK1B    340  APKARKYFDKL-PEGTWTVKKNKDLKKDYKVPGTRRLHEVLGVETGGPGGRRGGEQGHSP
zDYRK1B    340  APKARKYFDKL-SDGLWTVKKNKDIKKEYKPPATRRLHEILGVETGGPGGRRAGEQGHAP
 MNB       393  AHKTRKFFDKIVADGSYVLKKNQNGRK-YKPPGSRKLHDILGVETGGPGGRRLDEPGHSV

                ------------------ C-lobe ---------|
rDyrk1A    347  ADYLKFKDLILRMLDYDPKTRIQPYYALQHSFFKKTADEGTNTSNS---VSTSPAM
zDYRK1Aa   451  ADYLKFKDLILRMLDYDPKTRIQPYYALQHSFFKKTADEGTNTSSS---VSTSPAL
hDYRK1B    399  ADYLRFQDLVLRMLEYEPAARISPLGALQHGFFRRTADEATNTGPAGSSASTSPAP
xDYRK1B    399  SDYLKFKDLILRMLDYDPKTRITPFYALQHNFFKKTTDEGTNTSNS---NSTSPAM
zDYRK1B    399  CDYLKFKDLILRMLDYDPKTRITPFYALQHNFFKKTTDEGTNTSSS---TSTSPAM
 Mnb       452  SDYLKFKDLILRMLDFDPKTRVTPYYALQHNFFKRTADEATNTSGA---GATANAG
```

Fig. S1. Sequence alignment of the DYRK1 orthologs in Fig. 3.
Only the conserved sequences of the N-terminal region and the catalytic domain are shown. Positions with identical or similar amino acids in at least three sequences are highlighted in

black and grey, respectively. Amino acids that are unique in human DYRK1B and conserved in the other class 1 DYRKs are shown in red. Structural (sub-) domains and motifs are labeled on top of the alignment. NLS, nuclear localization signal.

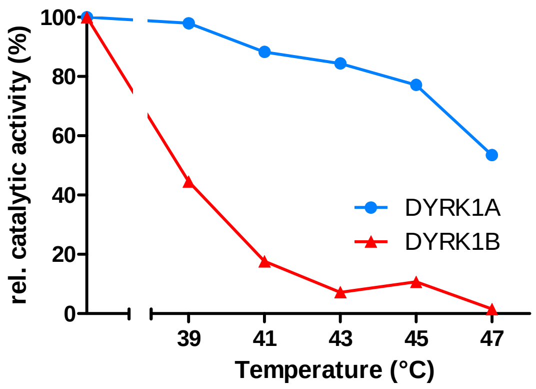

Figure S2: Differential thermal stability of GST-DYRK1AΔC and GST-DYRK1BΔC
The experiment shown in Fig. S2 was reproduced with independent preparations of the GST fusion proteins. Proteins were subjected to a heat shock (3.5 min) at variable temperatures before kinase assay. Catalytic activity was normalized to the activity of a control sample that was not exposed to heat shock.

Table S1: Sequences used for the construction of the dendrogram in Fig. 3A

Designation	Species	NCBI accession number	Designation	Species	NCBI accession number
**hDYRK1A**	*Homo sapiens*	NP_001387.2	**xDYRK1B**	*Xenopus laevis*	XP_018082132.1
**xDYRK1A.S**	*Xenopus laevis*	NP_001156669.1	**zDYRK1B**	*Danio rerio*	XP_005158274.1
**xDYRK1A.L**	*Xenopus laevis*	XP_018101384.1	**DYRK1**	*Dictyostelium discoideum*	XP_642598.1
**zDYRK1Ab**	*Danio rerio*	NP_001334760.1	**DYRK1**	*Ciona intestinalis*	XP_018669282.1
**zDYRK1Aa**	*Danio rerio*	NP_001334668.1	**MNB**	*Drosophila melanogaster*	NP_728104.1
**hDYRK1B**	*Homo sapiens*	NP_004705.1	**MBK-1**	*Caenorhabditis elegans*	CAA93756.2

Uncropped blots to Fig. 1-6

Uncropped blots (Fig. 1)

Fig. 1C

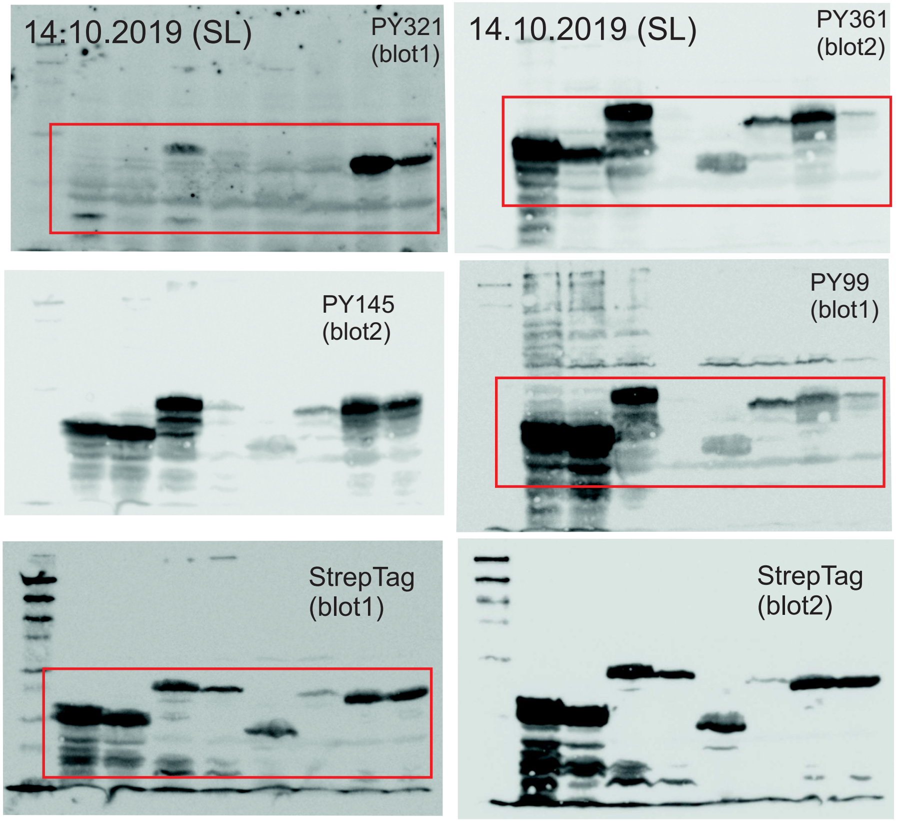

Fig. 1D 37°C

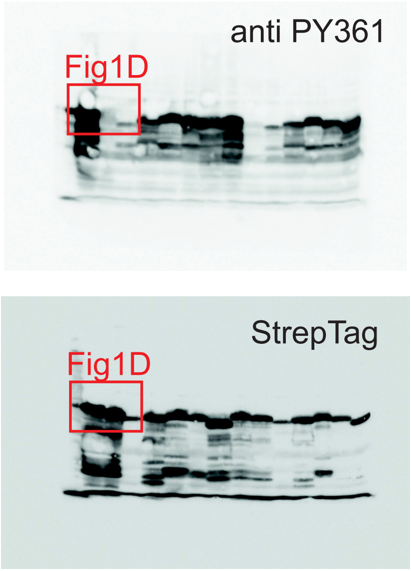

Fig. 1D RT

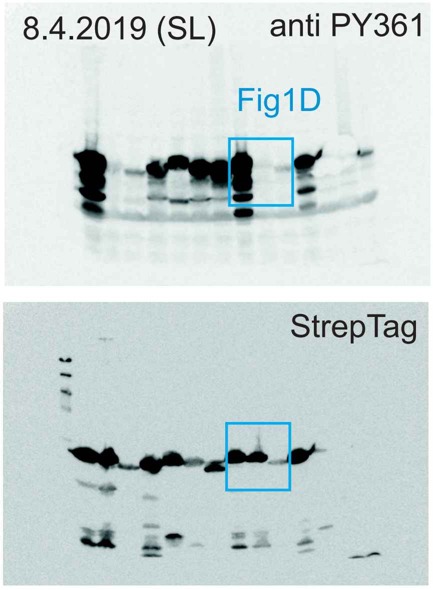

uncropped blots (Fig. 2A)

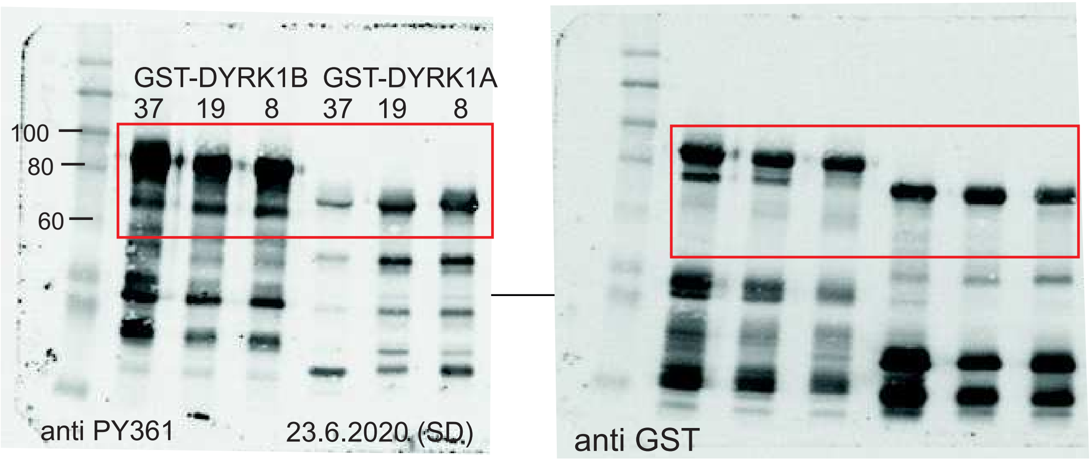

uncropped blots (Fig. 3B)

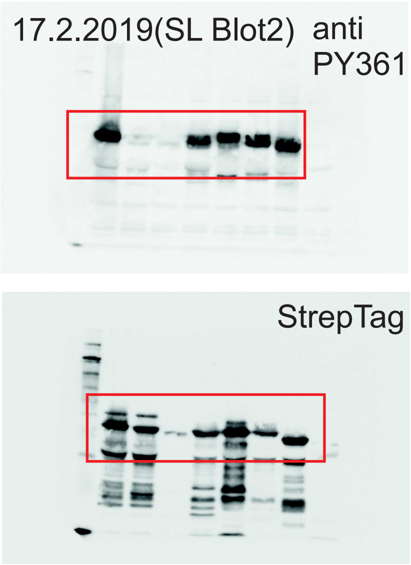

uncropped blots (Fig. 3D)

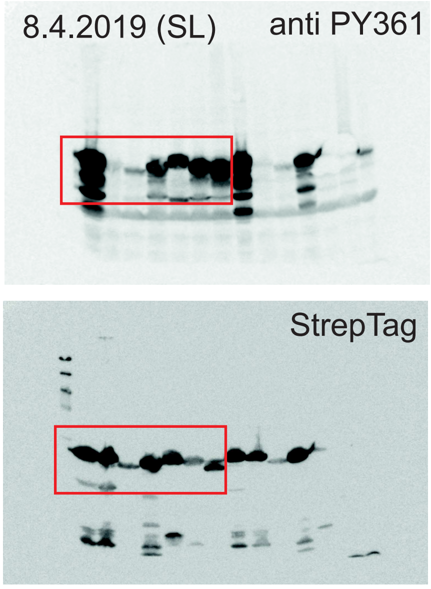

uncropped blots (Fig.4B)

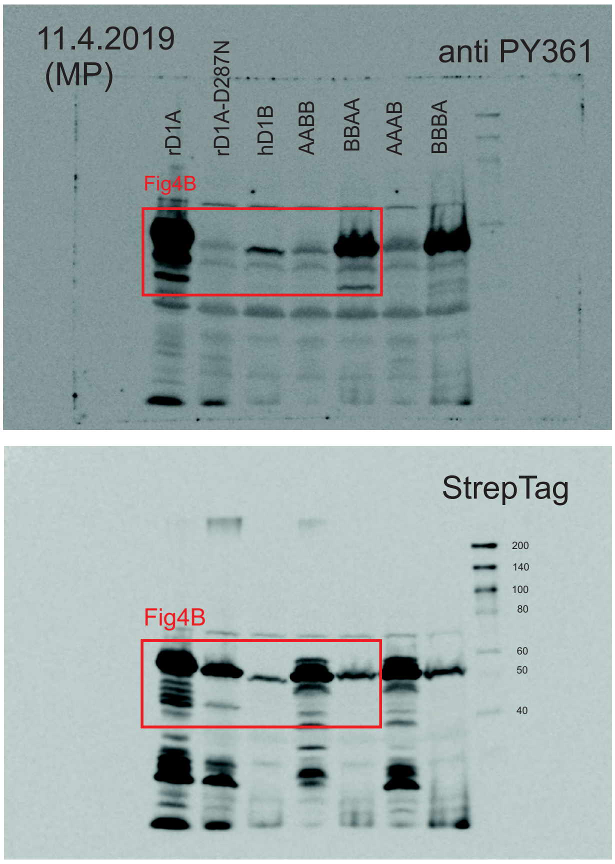

uncropped blots (Fig.4D)

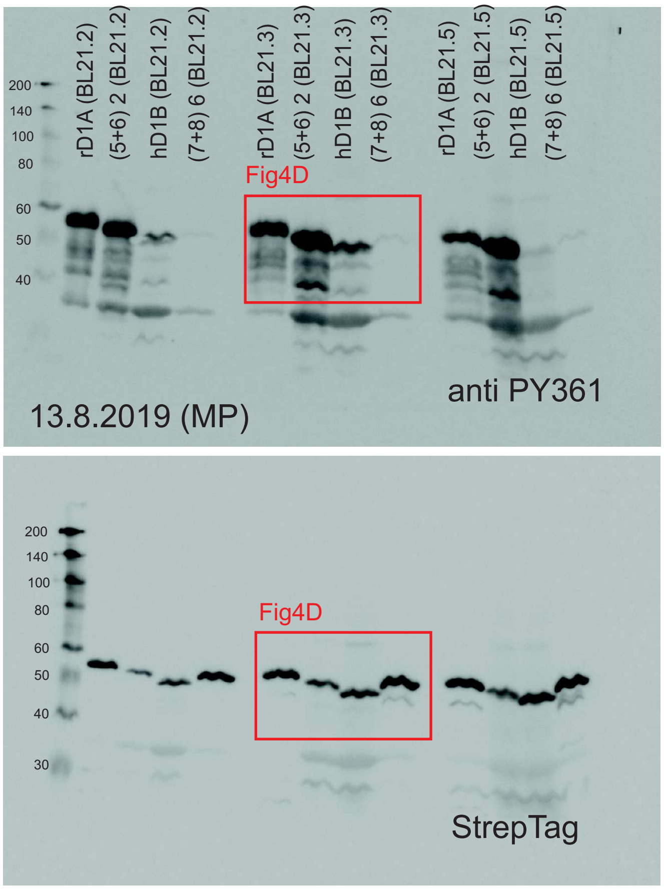

uncropped blots (Fig. 4F)

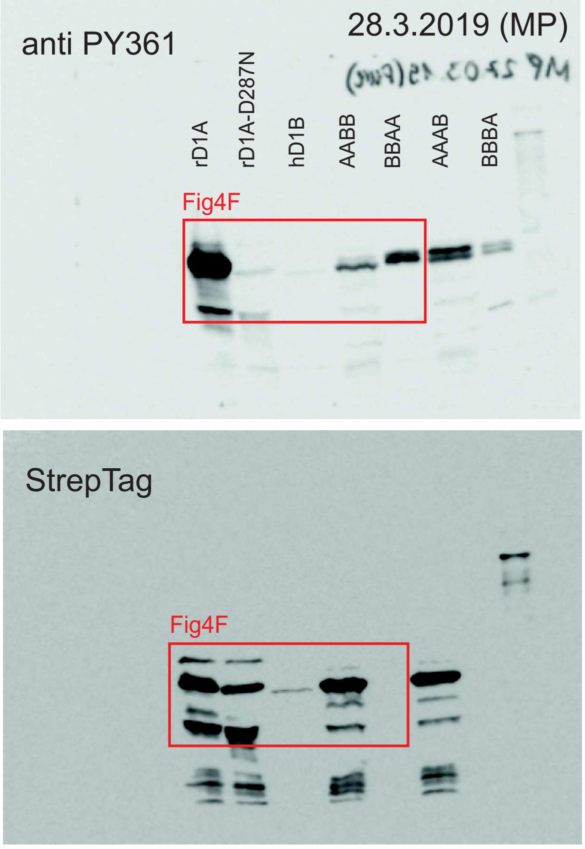

uncropped blots (Fig.5C)

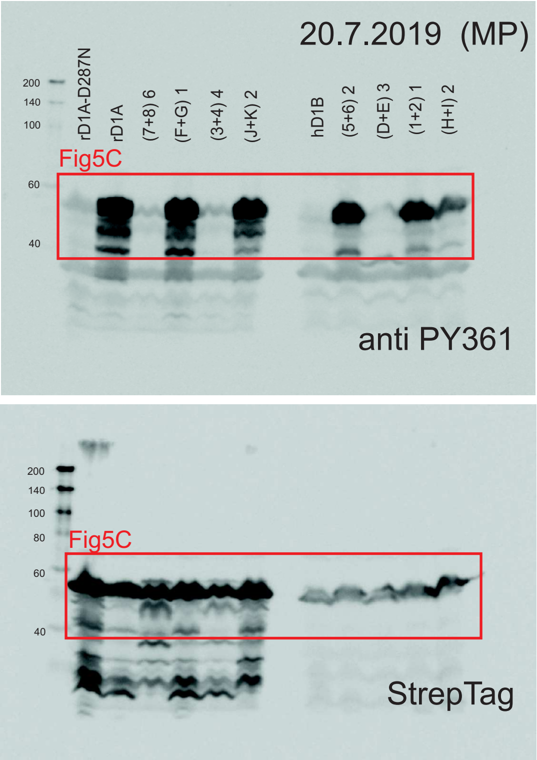

uncropped blots (Fig.5F)

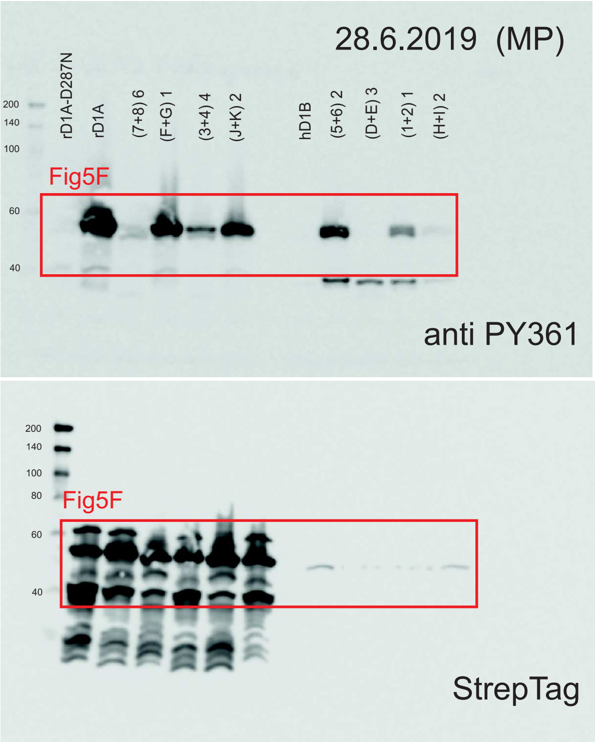

uncropped blots (Fig.6A)

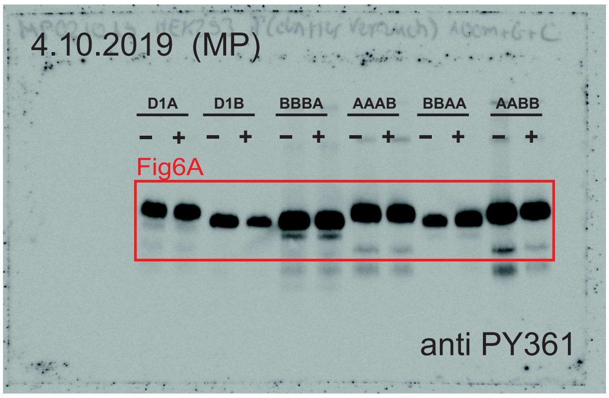

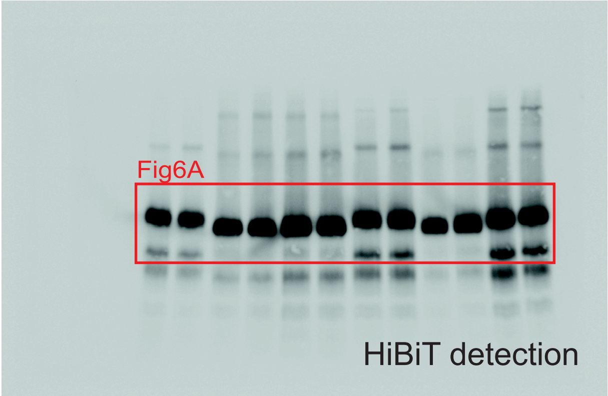

uncropped blot (Fig. 6C)

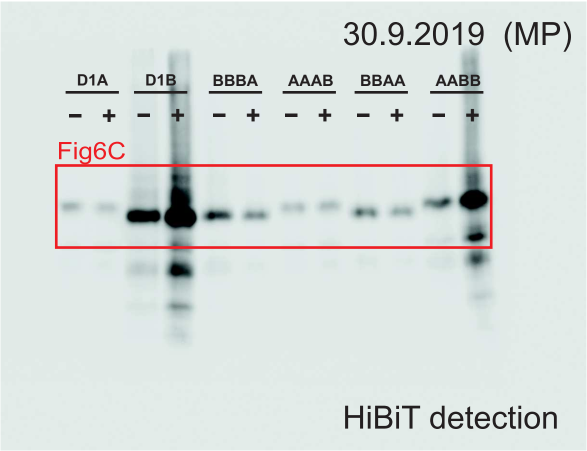

Supplement: Supplementary file 1 — Supplementary Information. [file 41598_2022_6423_MOESM1_ESM.pdf]
